# Identification of African swine fever virus-like elements in the soft tick genome provides insights into the virus’ evolution

**DOI:** 10.1186/s12915-020-00865-6

**Published:** 2020-10-08

**Authors:** Jan H. Forth, Leonie F. Forth, Samantha Lycett, Lesley Bell-Sakyi, Günther M. Keil, Sandra Blome, Sébastien Calvignac-Spencer, Antje Wissgott, Johannes Krause, Dirk Höper, Helge Kampen, Martin Beer

**Affiliations:** 1 Friedrich-Loeffler-Institut, Federal Research Institute for Animal Health, Südufer 10, 17493 Greifswald-Insel Riems, Germany; 2 The Roslin Institute, University of Edinburgh, Easter Bush, Midlothian, EH25 9RG Scotland, UK; 3 Institute of Infection, Veterinary and Ecological Sciences, University of Liverpool, 146 Brownlow Hill, Liverpool, L3 5RF UK; 4 Robert Koch-Institut Berlin, Seestraße 10, 13353 Berlin, Germany; 5 Max Planck Institute for the Science of Human History, Kahlaische Str. 10, 07745 Jena, Germany

**Keywords:** African swine fever virus, Coevolution, Endogenous viral element, Tick cell line, RNA interference, ASFLI-element

## Abstract

**Background:**

African swine fever virus (ASFV) is a most devastating pathogen affecting swine. In 2007, ASFV was introduced into Eastern Europe where it continuously circulates and recently reached Western Europe and Asia, leading to a socio-economic crisis of global proportion. In Africa, where ASFV was first described in 1921, it is transmitted between warthogs and soft ticks of the genus *Ornithodoros* in a so-called sylvatic cycle. However, analyses into this virus’ evolution are aggravated by the absence of any closely related viruses. Even ancient endogenous viral elements, viral sequences integrated into a host’s genome many thousand years ago that have proven extremely valuable to analyse virus evolution, remain to be identified. Therefore, the evolution of ASFV, the only known DNA virus transmitted by arthropods, remains a mystery.

**Results:**

For the identification of ASFV-like sequences, we sequenced DNA from different recent *Ornithodoros* tick species, e.g. *O. moubata* and *O. porcinus*, *O. moubata* tick cells and also 100-year-old *O. moubata* and *O. porcinus* ticks using high-throughput sequencing. We used BLAST analyses for the identification of ASFV-like sequences and further analysed the data through phylogenetic reconstruction and molecular clock analyses. In addition, we performed tick infection experiments as well as additional small RNA sequencing of *O. moubata* and *O. porcinus* soft ticks.

**Conclusion:**

Here, we show that soft ticks of the *Ornithodoros moubata* group, the natural arthropod vector of ASFV, harbour African swine fever virus-like integrated (ASFLI) elements corresponding to up to 10% (over 20 kb) of the ASFV genome. Through orthologous dating and molecular clock analyses, we provide data suggesting that integration could have occurred over 1.47 million years ago. Furthermore, we provide data showing ASFLI-element specific siRNA and piRNA in ticks and tick cells allowing for speculations on a possible role of ASFLI-elements in RNA interference-based protection against ASFV in ticks. We suggest that these elements, shaped through many years of co-evolution, could be part of an evolutionary virus-vector ‘arms race’, a finding that has not only high impact on our understanding of the co-evolution of viruses with their hosts but also provides a glimpse into the evolution of ASFV.

## Background

The pandemic spread of viral pathogens affecting humankind has been recognised for many years as one of the most dangerous scenarios leading to crises of global proportion. Furthermore, the ‘One Health’ concept of today is arguing that pathogens affecting animals or plants may also have a major impact on the human population [1, 2]. One recent example of such interrelations with an extreme global socio-economic impact is the unprecedented pandemic spread of African swine fever virus (ASFV), one of the most devastating viral diseases of animals [3].

ASFV, a double-stranded DNA (dsDNA) virus, can infect all kinds of suids and leads to a multi-systemic disease, African swine fever (ASF), in non-natural suid hosts with clinical signs of a viral haemorrhagic fever characterised by morbidity and case fatality rates of up to 100% [4, 5]. Despite almost 100 years of intensive research and the occurrence on four continents [6], neither a vaccine nor any treatment is available. Therefore, the introduction of ASFV into the wild boar populations of Eastern Europe in 2007 has led to an unprecedented epidemiological situation that culminated in the introduction of ASFV into Western Europe and Asia [7–9]. Especially in China, the largest producer of pork in the world, extreme socio-economic consequences can be observed, impacting not only Asia, but affecting the global agriculture and food industry and shifting entire markets [3, 10].

Nevertheless, very little is known about ASFV evolution. ASFV appears to be the only member of its genus (*Asfivirus*) and family (*Asfarviridae*) and is the only known DNA arbovirus [4]. First described from, and endemic in, sub-Saharan Africa [11], ASFV is transmitted in a sylvatic cycle between soft ticks of the *Ornithodoros moubata* complex and indigenous wild pigs such as African warthogs (*Phacochoerus africanus*) [12]. Both groups of natural hosts are well adapted to an ASFV infection and, although persistently infected [12], show no obvious pathological signs, which has led to the hypothesis that—similar to other arboviruses—ASFV and its hosts have undergone a long time of co-evolution. Since viruses leave no fossil records, analyses into the evolution of ASFV have been restricted to highly similar genotypes of which only very few whole-genome sequences are available [4, 13]. However, with the recent advances in sequencing technologies, novel ways of analysing virus evolution have been discovered, with one of those being the analysis of endogenous viral elements (EVE)—ancient viral sequences integrated into the host genome [14].

In our study, we identified African swine fever virus-like integrated (ASFLI)-elements in the uncharacterised genome of *O. moubata* complex soft ticks. We show ASFLI-elements in tick cell lines [15], *O. moubata* and *O. porcinus* soft ticks, and in museum-stored *Ornithodoros* ticks collected about 100 years ago in the field in East Africa, allowing for phylogenetic reconstruction and orthologous dating. To evaluate whether the ASFLI-elements retained a function, we conducted tick infection experiments, transcriptome analyses and small RNA sequencing and expressed a highly conserved ASFV-like gene from the tick genome for further characterisation.

## Results

### Evidence of ASFLI-elements in the *O. moubata* tick cell genome

In order to test for the presence of either novel DNA viruses or integrated viral elements, we sequenced DNA from the *O. moubata* cell line OME/CTVM21 [15] and combined reads from different sequencing platforms. In detail, 14.9 million paired-end reads with 300-bp read length (Illumina MiSeq) and 277 million single-end reads with 50-bp read length (Illumina HiSeq) were combined with about 500,000 3rd-generation sequencing single-molecule, ultra-long reads (MinION) (Additional file 1: Table S1).

**Fig. 1:**
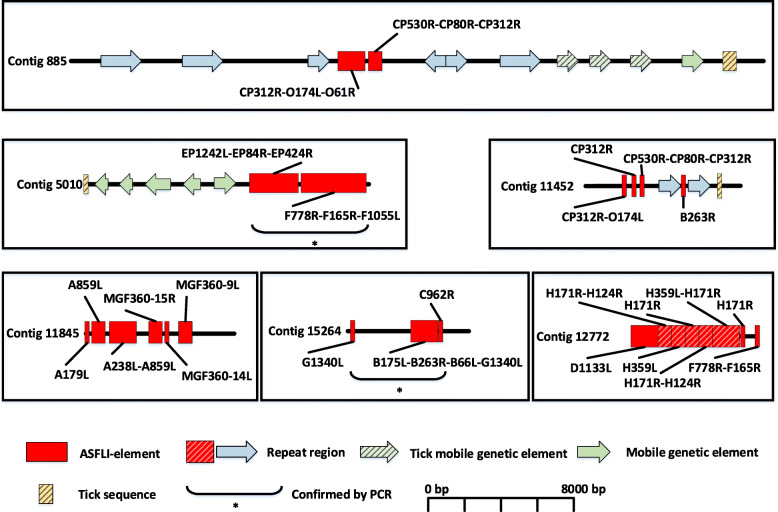
Integration sites of ASFLI-elements in the genome of the *Ornithodoros moubata* cell line OME/CTVM21 genome. Exemplary SPAdes-assembled contigs showing ASFLI-elements (red) adjacent to tick sequences (yellow-striped), genes from mobile genetic elements (green and green striped arrows) and repeat regions (blue arrow, red-striped) identified by BLASTn and BLASTp. Repeat regions refer to repetitive regions in the tick genome as identified by the Geneious ‘Find repeat’ option. They show a nucleotide sequence identity of > 95%. PCR and subsequent sequencing validated the assembly for two major ASFLI-elements (*)

**Fig. 2:**
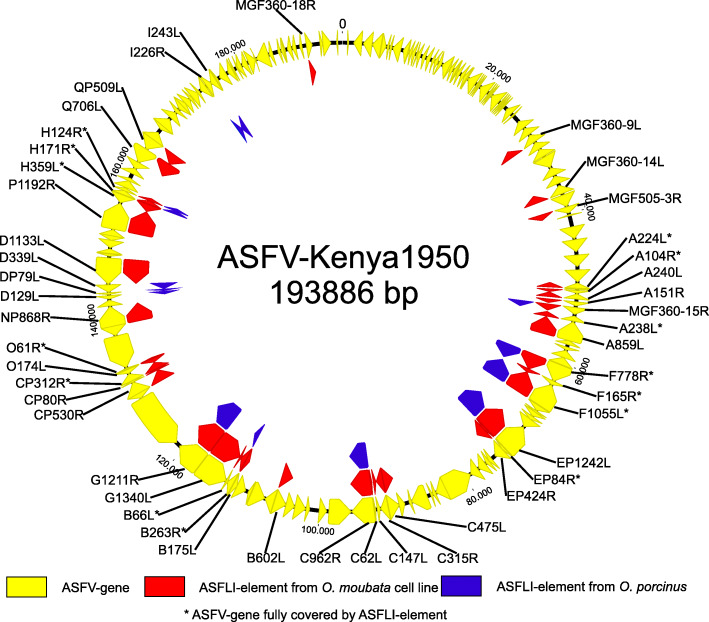
ASFLI-elements and homologous ASFV genes detected in *Ornithodoros moubata* tick cell lines and *Ornithodoros porcinus* ticks. ASFLI-elements from *O. moubata* (red) and *O. porcinus* (blue) were identified by BLASTn through their homologies to 32 partial and 14 complete (*) ASFV genes (yellow) distributed over the entire ASFV-Kenya 1950 (Acc. No. AY161360) core genome. For better visualisation, the viral genome is displayed as a circle

Using the SPAdes v3.11.1 software [16] for the assembly and BLASTn for the detection of viral sequences, we identified 34 contigs with lengths between 336 and 85,582 bp containing ASFLI-elements (Fig. 1 and Additional files 2, 3 and 4: Tables S2-S4). However, we detected truncated ASFV-like ORFs as well as sequence duplications and reorganisations when comparing ASFLI-elements with the homologous ASFV genes and the ASFV genome organisation (Figs. 1 and 2 and Additional files 3 and 4: Tables S3 and S4). For validation of the assembly, two large ASFLI-elements (6.8 kb and 4.2 kb) were amplified by PCR and subsequently sequenced in a shotgun sequencing approach (Illumina MiSeq) followed by assembly using Newbler 3.0 (Roche) and alignment to the previously assembled SPAdes contigs using MAFFT v7.388 in Geneious (Fig. 1). The BLASTn search as well as the analysis of the > 500-bp ORFs by BLASTp resulted in the identification of contigs showing both integrated viral elements and sequences with similarity to the genome of the black-legged tick *Ixodes scapularis*, the only tick species with a fully sequenced and partially annotated genome [17]. Moreover, contigs were identified that cover multiple ORFs with similarity to mobile genetic elements (Fig. 1 and Additional files 3 and 4: Tables S3 and S4). Altogether, we were able to identify ASFLI-elements homologous to 46 ASFV genes, covering 14 genes completely and 32 genes partially, with identities to recent ASFV sequences of up to 86.6% (Fig. 2 and Additional files 3 and 4: Tables S3 and S4).

### Phylogenetic analysis shows ASFLI-elements are close relatives of ASFV sequences

**Fig. 3:**
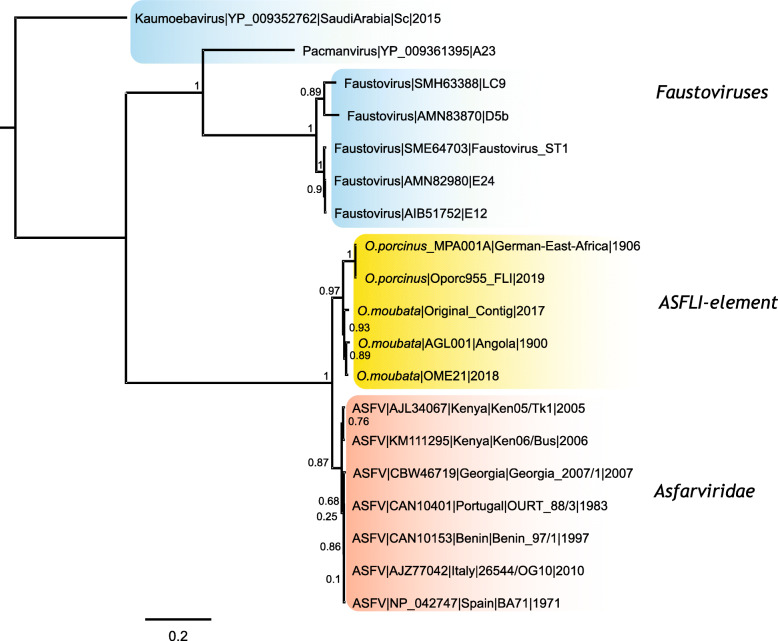
Phylogeny of ASFLI-pEP1242L and homologues. Maximum likelihood tree (JTT+G_4_) showing ASFLI-elements, ASFV sequences and NCLDV background protein sequences for EP1242L (DNA-directed RNA polymerase subunit 2) and analogues. Statistical support of 100 bootstraps is indicated at the nodes. The scale bar represents the number of substitutions per site

In order to place the ASFLI-elements in the context of other nucleocytoplasmic large DNA viruses (NCLDV), the ASFLI-elements corresponding to ASFV proteins pF1055L (Helicase) and pEP1242L (RNA polymerase subunit 2) were chosen because (a) they were present in the samples and quite complete, (b) are quite long and (c) these genes are present in the Megavirales background. Both were aligned with homologous NCLDV amino acid sequences including sequences of ASFV (Additional file 5: Supplementary Appendix). The amino acid sequence trees of the NCLDV are diverse, but both ASFLI-elements, pF1055L (Fig. 3) and pEP1242L (Additional file 5: Supplementary Appendix), group with the ASFV amino acid sequences, thus forming well-supported monophyletic clades (bootstrap value = 1). The ASFLI-elements are, however, placed as a close outgroup to the other ASFV sequences (Fig. 3 and Additional file 5: Supplementary Appendix).

### ASFLI-elements are present in recently sampled *O. moubata*, *O. porcinus* and approx. 100-year-old *O. moubata* and *O. porcinus* field-collected ticks from Africa

To evaluate if the integration of the ASFLI-elements is specific for the *O. moubata* cell line OME/CTVM21, we tested all six available *Ornithodoros* cell lines as well as laboratory-reared *O. moubata* ticks from various origins using NGS and quantitative PCR (qPCR) for ASFLI-elements. Furthermore, we analysed *O. porcinus* ticks collected from the field in Kenya in 2017 and from a French laboratory-reared colony, *Ornithodoros erraticus* field ticks from Portugal and *Ornithodoros savignyi* from Nigeria. While all tick cell lines and the *O. moubata* ticks tested positive for ASFLI-elements, *O. porcinus*, *O. savignyi* and *O. erraticus* (Additional file 6: Table S5) tested negative. However, by sequencing DNA from the *O. porcinus* ticks (Additional file 1: Table S1) and assembly, we were able to identify 18 contigs containing ASFLI-elements with lengths over 150 bp which were, due to the differences in the nucleotide sequence identity to the *O. moubata* ASFLI-elements, not detected by qPCR. In total, these *O. porcinus* ASFLI-elements relate to 14 ASFV genes (Fig. 2 and Additional files 1 and 7: Tables S1 and S6) and for five, corresponding ASFLI-elements were only detected in *O. porcinus* (Fig. 2 and Additional files 1 and 7: Tables S1 and S6). One possible orthologous element, including the ASFV-like EP1242L gene, was discovered in *O. moubata* and *O. porcinus* (98.5% nucleotide sequence identity flanking a piggyBac transposable element with 90.3% nucleotide sequence identity) (Additional file 8: Figure S1).

**Fig. 4:**
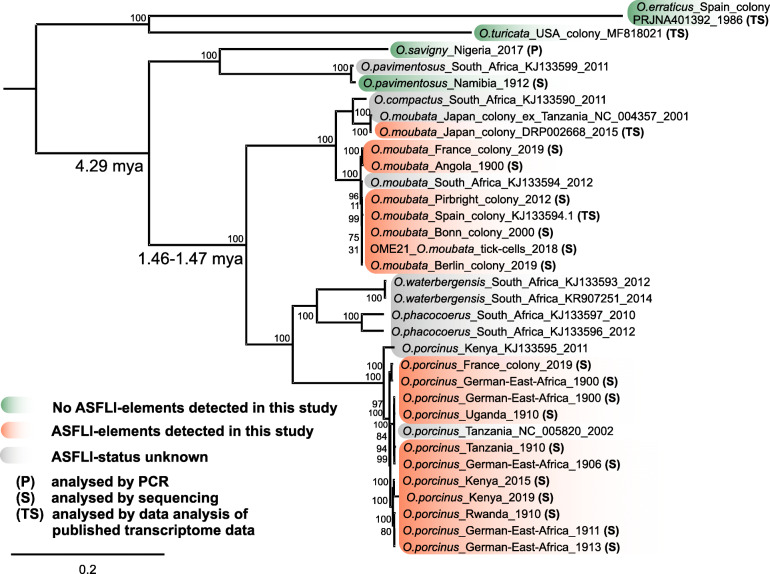
*Ornithodoros* phylogeny and orthologous dating of ASFLI-element integration. A maximum-likelihood (ML) tree was constructed using IQ-TREE v1.6.5 with standard model selection, resulting in the best-fit model TIM2+F+R3 (AC=AT, CG=GT and unequal base frequencies + empirical base frequencies + FreeRate model with 3 categories) based on MAFFT v7.388 aligned full-length mitochondrial sequences from *Ornithodoros* ticks. Statistical support of 10,000 ultrafast bootstraps is indicated at the nodes. Taxon names include, where available, tick species designation, country of origin, library number, INSDC accession number and sampling date. Clock data on tick species divergence was included from the literature [20]. The scale bar represents the number of substitutions per site

Through the analysis of published transcriptome data from *Ornithodoros* ticks, we identified ASFLI-element-specific RNA in *O. moubata* ticks from laboratory colonies from Spain [18] and Japan [19] (Fig. 4 and Additional files 1 and 2: Tables S1 and S2), while in pertinent data from *O. erraticus* [21] and *Ornithodoros turicata* (PRJNA447876), no ASFLI-specific RNA was detected (Fig. 4). Furthermore, we examined nine *Ornithodoros* specimens collected between 1906 and 1913 in the former German colonies of East and Southern Africa that had been stored in ethanol at the Berlin Museum for Natural History (MfN) by sequencing (Fig. 4 and Additional files 1 and 9: Tables S1 and S7). In all libraries relating to *O. moubata* and *O. porcinus* ticks, we detected ASFLI-element-specific reads or even contigs while in one library relating to *Ornithodoros pavimentosus*, no ASFLI-element-specific DNA could be detected (Fig. 4, Additional file 9: Table S7).

### Phylogenetic reconstruction using full-length mitochondrial genomes of soft ticks reveals a possible integration of an ASFLI-element might have occurred over 1.46–1.47 million years ago (mya)

To assess the phylogenetic relationship between the different tick species carrying ASFLI-elements and perform orthologous dating, we generated full-length mitochondrial genomes from the tick sequence data generated in this study (Additional file 1: Table S1). These genomes were aligned together with additional tick mitochondrial genome sequences from the literature [20] using MAFFT v7.388 in Geneious, and a phylogenetic tree (Fig. 4) was constructed using IQ-TREE v1.6.5. Molecular clock data from the literature on tick species divergence, which was calibrated using fossil data [20], suggests that the *O. moubata* group including *O. moubata*, *O. porcinus*, *O. compactus*, *O. waterbergensis* and *O. phacocoerus* originated 4.29 mya and that *O. moubata* and *O. porcinus* probably diverged 1.46–1.47 mya [20]. We detected a major ASFLI-element flanking a mobile genetic element showing the same orientation and high nucleotide sequence identities in both *O. moubata* and *O. porcinus* (Additional file 8: Figure S1). Since no more comparable ASFLI-element containing loci could be defined, we analysed if multiple copies of this mobile genetic element could exist per genome, e.g. if this combination of ASFLI-element and mobile genetic element might have happened independently by chance. Therefore, we mapped DNA sequencing data from the libraries lib02151-2 and lib02339 against Contig 22316_OME and lib03101-2 against Contig 955_OPF using Bowtie2 with default parameters in the ‘very sensitive’ mode. In the case that multiple copies of the transposable element exist, a strong increase in coverage would be expected when compared to the coverage of the ASFLI-element. As displayed (Additional file 5: Figure S5), no significant difference could be detected in *O. moubata* and in *O. porcinus*. However, a BLASTn search of the transposable element sequence against the SPAdes assembled contigs of *O.* moubata and *O. porcinus* using only hits with an *e* value lower than 0.0001 revealed no additional contigs including this transposable elements nucleotide sequence. Therefore, we suggest that this ASFLI-element might have been integrated in a shared integration event that could have occurred before both species diverged 1.46–1.47 mya (Fig. 4). Because we did not discover any ASFLI-element-specific DNA in *O. pavimentosus* (Fig. 4), which diverged from *O. moubata* and *O. porcinus* around 4.29 mya, it could further be hypothesised that the maximum time to the integration is 4.29 mya.

### Molecular clock analyses using ASFLI-elements from different *Ornithodoros* species provide an estimate for a time to the most recent common ancestor consistent with orthologous dating

In addition to the mitochondrial genomes, we analysed the orthologous ASFLI-element containing ASFV-EP1242L from five soft tick specimens (three *O. moubata* and two *O. porcinus*). These sequences contained deletions and one insertion in comparison with the homologous ASFV-EP1242L. Maximum likelihood phylogenies from the corresponding amino acid sequences revealed that all samples clustered as an outgroup close to the *Asfarviridae* (Additional file 5: Supplementary Appendix). Due to missing data points over time that would be useful for calibration, inferring time-scaled phylogenies on the EP1242L containing ASFLI-element sequences from the soft tick samples was more challenging than for the mitochondrial genomes. However, by using a strict clock with molecular clock rate priors of 5e−7 to 1e−8 substitutions per site per year (commensurate with the expected substitution rate for host DNA), a time to most recent common ancestor (TMRCA) of 0.08 to 4.7 million years was inferred (Additional file 5: Supplementary Appendix). Although the confidence intervals on these TMRCA are very wide, the TMRCA of the EP1242L ASFLI-element is consistent with the overall integration estimate of 1.46–4.29 million years obtained from orthologous dating using the host mitochondrial genomes (Additional file 5: Supplementary Appendix).

### *Ornithodoros* tick species and tick cell lines show differences in the infectability with various ASFV genotype isolates

Since we detected different repertoires of ASFLI-elements in *O. porcinus* and *O. moubata* (Fig. 2), we investigated the differences in the infectability of both tick species with different ASFV strains. Therefore, we infected *O. porcinus* ticks from Kenya and *O. moubata* ticks from the Berlin colony by membrane-feeding with 1 × 10^4^ and 1 × 10^6^ haemadsorbing units (HAU)/ml of the ASFV ken.rie1 P72 genotype X strain that was originally isolated from a Kenyan *O. porcinus* tick. To analyse the influence of different ASFV genotypes on the infection rate of the ticks, *O. porcinus* and *O. moubata* ticks were furthermore infected with 1 × 10^5^ HAU/ml of an ASFV-Ken06.bus genotype IX strain or with 1 × 10^4^ HAU/ml of an ASFV-Sardinia genotype I isolate. As a marker for a successful early phase and transition into the late phase of infection, all tick specimens were tested for the accumulation of the late-expressed *p72*-specific viral transcripts by (RT-qPCR), and the virus titre of the blood-virus solution used for oral infection was determined by titration on porcine macrophages.

**Fig. 5:**
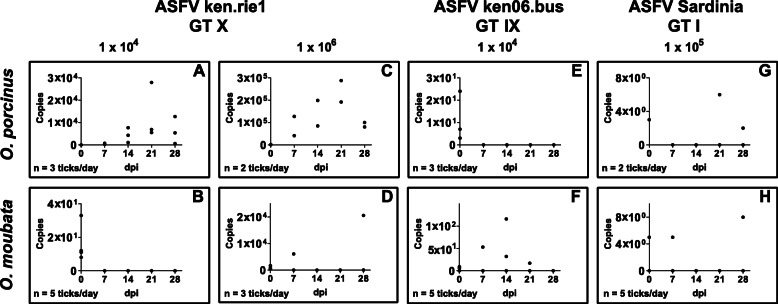
RT-qPCR results of *Ornithodoros* spp. ticks experimentally infected with different ASFV genotypes. Shown are ASFV-P72 transcript-specific copy numbers of third nymphal stage ticks fed with defibrinated pig blood containing either 1 × 10^4^ HAU/ml or 1 × 10^6^ HAU/ml ASFV ken.rie1 (GT X) (**a–d**), 1 × 10^5^ HAU/ml ASFV Ken06.bus (GT IX) (**e**, **f**) or 1 × 10^4^ HAU/ml ASFV Sardinia (GT I) (**g**, **h**). Due to the limited number of field ticks available and ticks feeding under artificial conditions, fifteen ticks were collected in each of three experiments (three per time point) (**a**, **d**, **e**), ten in two experiments (two per time point) (**c**, **g**) and twenty-five individuals in each of three experiments (five per time point) (**b**, **f**, **h**) (Additional file 10: Table S8). All ticks were stored at − 80 °C prior to individual RNA extraction and RT-qPCR analysis

In the initial experiments (Fig. 5a, c), all *O. porcinus* ticks showed a clear accumulation of viral RNA while from the *O. moubata* ticks, only two accumulated viral transcripts after infection with the higher virus dose (Fig. 5b, d and Additional file 10: Table S8). While among the ticks infected with ASFV Ken06.bus, a minimal *p72* transcript accumulation was detected only in four *O. moubata* ticks (Fig. 5e, f), two *O. moubata* and one *O. porcinus* tick infected with ASFV Sardinia showed a minimal increase of *p72* transcripts (Fig. 5g, h). Furthermore, infection of the tick cell lines OME/CTVM21, OME/CTVM22, OME/CTVM24 and OME/CTVM27 with ASFV ken.rie1 did not result in any detectable viral transcription (data not shown).

### RNA sequencing demonstrates ASFLI-element-specific mRNA—small-interfering and piwi-interacting RNAs in tick cells

**Fig. 6:**
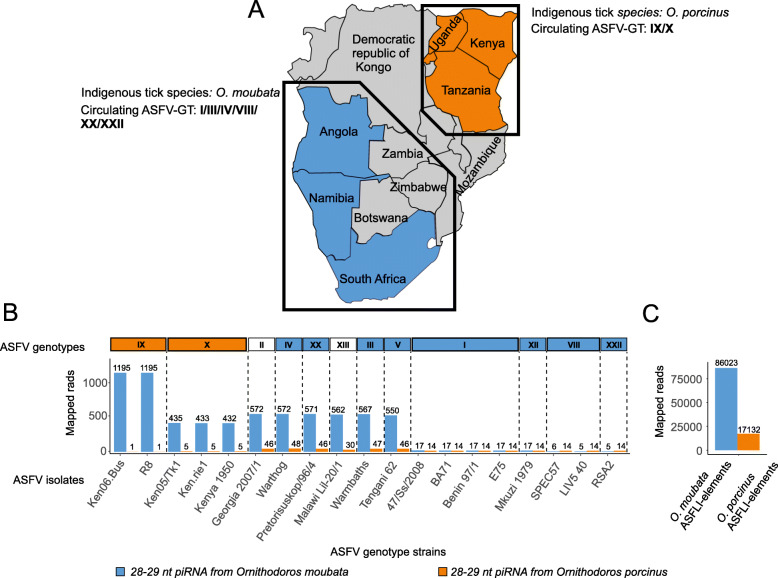
Differences in *O. moubata* and *O. porcinus* piRNA identity with specific ASFV genotypes and ASFLI-elements. piRNA (28-29 nt) reads from *O. moubata* (originating from southern Africa) and *O. porcinus* ticks (originating from East Africa) (**a**) were mapped against available ASFV whole-genome sequences from different genotypes with Bowtie (**b**). While the lowest number of mapped *O. moubata* piRNA reads was observed for ASFV genotypes shown to infect *O. moubata* (ASFV-GT I, XII, VIII, XXII), the lowest number of *O. porcinus* piRNA reads mapped to ASFV genotype X able to infect *O. porcinus* (**b**). With up to 100% sequence identity, 86.023 (*O. moubata*) and 17.132 (*O. porcinus*) reads mapped to the ASFLI-elements (**c**)

Interference with small RNAs (RNAi), e.g. small-interfering RNA (siRNA) or piwi-interacting RNA (piRNA), could be responsible for the observed differences in ASFV infection rates of the *Ornithodoros* ticks (as shown in insects [22]); we therefore investigated hypothetical ASFLI-element-specific small RNAs in the ticks and performed small RNA sequencing (Additional files 1, 11, 12, 13, 14 and 15: Figure S2-S4 and Tables S1 and S9). The distribution of the read lengths showed a distinct bimodal distribution in all libraries with one peak at 22 nt, representing siRNAs, and one at 28–29 nt, representing piRNAs (as shown by the typical U-bias at the 5′-end) (Additional files 11 and 15: Figure S2 and Table S9) [23]. This is in accordance with small RNA sequencing data from hard ticks [24, 25]. By mapping the resulting data (siRNA and piRNA fractions) against the ASFLI-element-containing contigs, we identified siRNAs and piRNAs homologous to different ASFLI-elements (Additional files 12 and 13: Figure S3 and S4). Sequence analysis of these piRNAs (mapped to ASFLI-elements from both strands) also revealed a clear 5′-U bias (Additional file 11: Figure S2C). While for some elements, none or only single homologous siRNA/piRNA molecules were identified, elements related to the ASFV genes EP1242L and F778R showed multiple corresponding siRNAs/piRNAs. Furthermore, sequence analysis of piRNA mapped to ASFLI-elements from *O. moubata* and *O. porcinus* revealed piRNAs with ping-pong signatures indicative of ping-pong cycle amplification (Additional files 16 and 17: Table S10 and S11) [26]. Following the mapping of all siRNAs and piRNAs against available ASFV whole-genome sequences from different viral genotypes, we observed matching RNAs but identified differences in the number of mapped reads from the different tick species. While the highest number of siRNA and piRNA from *O. moubata* mapped to ASFV genotypes IX and X, piRNA from *O. porcinus* mapped best to ASFV genotypes II, III, IV and V (Fig. 6 and Additional files 12, 13, 14 and 15: Figure S3-S5 and Table S9), the siRNA fraction of *O. porcinus*, however, mapped best to genotype IX and X isolates. The mapped piRNA reads also showed a clear 5′-U-bias (Additional file 11: Figure S2C). In addition to the small RNA, we detected ASFLI-element-specific mRNA in the OME/CTVM21 cells as well as in *O. moubata* transcriptome data from the literature (Additional files 1, 2: Tables S1-S2).

### The reconstructed ASFLI-A104R protein is highly similar to its ASFV homologue but is not expressed in tick cell lines

Among the complete ASFLI-element genes discovered by NGS, ASFLI-A104R is one of the most conserved with nucleotide sequence identities of 83.0–85.0% and amino acid sequence identities of 94.0–95.0%. However, the insertion of an ‘A’ nucleotide in a homopolymer region of eight ‘A’ nucleotides, starting at position 9 of the gene, leads to a frameshift and stop codon integration. Since no small RNA molecules matching ASFLI-A104R were identified, we investigated whether the ASFLI-A104R protein is expressed in the cultured tick cells. We repaired the alA104R-ORF in silico by removing the additional ‘A’, expressed the synthetic gene as both a native protein and a flag-tagged variant in *Escherichia coli* and raised rabbit sera against the purified alA104R proteins. Using Western blot analysis, we observed specific signals for the native protein as well as for the flag-tagged alA104R protein, and also for the ASFV-Kenya 1033 protein. However, no ASFLI- A104R-specific signals were obtained with protein extracts from the different tick cell lines (Additional file 18: Figure S6).

## Discussion

We here report the existence of endogenous ASFLI-elements in the genome of *O. moubata* complex soft ticks. The detection of these ancient integrated sequences will allow us, for the first time, to gain deeper insights into the evolution of ASFV, one of the most dangerous animal pathogens known to date, and into the role of endogenous viral elements in the co-evolution of ticks and viruses.

Generally, very little is known about tick genome organisation or how and how often viral elements become integrated into a tick genome [27] and for what reasons. We identified ORFs related to mobile genetic elements as well as repeat regions and possible target site duplications formed by the integration of mobile genetic elements adjacent to the ASFLI-elements. Therefore, it might be hypothesised that the integration of viral elements into large hyper-variable regions of the tick genome [17, 28–30] is assisted by the interaction with mobile genetic elements as discussed for endogenous Borna-like N (EBLN) elements [31] and various arthropod viruses [28, 30, 32, 33].

Further analyses showed that *O. moubata* and *O. porcinus* ticks, in addition to the *O. moubata* tick cell lines, harbour ASFLI-elements. This observation suggests the fixation of these elements in the *O. moubata* and *O. porcinus* genomes. While it remains unclear how the fixation might have occurred, the initial event leading to germline integration might be explained by the fact that ASFV infects the tick’s reproductive tissues in order to become transovarially and venereally transmitted. Furthermore, although ASFV replication takes place in the cytoplasm, viral mRNAs were demonstrated to enter the nucleus of infected Vero cells and primary porcine macrophages [34, 35], where they could have been integrated. However, more individual *O. moubata* and *O. porcinus* ticks from different geographical locations need to be analysed to proof the fixation of the ASFLI-elements in the *O. moubata* and *O. porcinus* genomes.

Although we detected matching ASFLI-elements in different *Ornithodoros* tick species, the occurrence of multiple integration events over time cannot be ruled out. However, the identification of a highly identical ASFLI-element including the ASFV-like EP1242L gene flanking a highly identical piggyback transposable element sequence in the same orientation in both *O. moubata* and *O. porcinus* might suggest that one integration event might have taken place over 1.46–1.47 million years ago. Since we were not able to detect ASFLI-elements in *Ornithodoros* species other than *O. moubata* and *O. porcinus*, we can further estimate a maximum time to the integration of 4.29 million years ago, when these species diverged. However, a more detailed analysis of *Ornithodoros* ticks in and outside of the *O. moubata* group including DNA sequencing are needed to further substantiate this assumption. Although the molecular clock analysis using the orthologous ASFLI-elements resulted in a TMRCA consistent in range with the orthologous dating, missing data through time hindered calibration, and thus, model selection was difficult and the confidence intervals are very wide. Therefore, and since the pattern and rate of substitutions of the ASFLI-elements could have been furthermore influenced by selection, the results should be interpreted with caution and more data on ASFLI-elements in different *Ornithodoros* tick species is needed to define the possible time point of integration more precisely. Whether the integration of non-retroviral sequences into a host genome is a directed process and how often it leads to improved fitness is controversially discussed [30, 36, 37]. However, protection against homologous viruses was demonstrated after integration of retroviral elements in mice and sheep [37] and is also discussed for EBLN elements [28, 31]. Furthermore, the transcriptional activity of EVEs and protection against viral infection was demonstrated to occur in crustaceans [30, 32, 33] and mosquitoes [38, 39].

In total, we identified ASFLI-elements in the genomes of *O. moubata* and *O. porcinus* ticks homologous to 46 ASFV genes; most of them represent genes with properties linked to viral DNA replication or RNA transcription. These observations may be explained by the fact that these genes in particular are highly conserved between viral isolates (90–100%); the genes coding for structural proteins are more variable; therefore, we cannot exclude that the latter, if also integrated, are simply not detectable by identity-based search methods like BLAST. However, the integration of major viral structure protein genes might have been harmful for the tick cell, so that they were just not maintained in the genome. Furthermore, it might be possible that that the integration of the conserved viral core genes, essential for the early phases of viral infection, is in particular linked to a beneficial function, such as protection by RNAi.

During infection experiments using *O. moubata*, *O. porcinus* and different ASFV genotypes, we observed differences in the ability of ASFV to infect the various *Ornithodoros* species. While a genotype X isolate from Kenya was able to replicate in *O. porcinus* ticks from Kenya, no infection was observed in *O. moubata* (phylogenetically closest to southern African ticks). However, when using a higher viral titre, successful infection could be observed in a small number of *O. moubata* ticks indicating that, in principle, infection is possible and that low-level infection might be blocked by a tick defence mechanism, such as RNAi.

Through the identification of ASFLI-element-specific siRNA and piRNA in the ticks and tick cells, we provide data suggesting that the ASFLI-elements could be involved in RNAi-based defence against ASFV infection in *Ornithodoros* ticks. Both siRNA and piRNA are known as major regulation factors of transcription, transposon suppression and defence against viral pathogens [40–43]. Although for ticks the known arsenal of antiviral mechanisms includes protection by RNAi [44], the role of EVEs in RNAi and protection against viral pathogens has never been reported.

The results of our infection experiments with different ASFV genotypes using the *O. porcinus* and *O. moubata* ticks are in agreement with observations from other studies in which clear differences were shown in tick infection rates using different ASFV genotype isolates [45–49]. Together with our data on ASFLI-element-specific siRNA and piRNA in these ticks, it could be hypothesised that the ASFLI-elements might play a role in determining which ASFV can infect which tick species. Therefore, it could be speculated that during millions of years of co-evolution, where ASFV hosts were regionally isolated by geographical factors (e.g. mountains, rivers), ASFV and its soft tick hosts have adapted to co-exist as displayed by the regional distribution of ASFV genotypes and the lack of any pathological findings in infected soft ticks, despite generalised virus infection and high viral titres. However, additional immunological or genetic factors determining which ASFV strain can infect a specific *Ornithodoros* tick species need to be considered, and more infection experiments using different *Ornithodoros* tick species and ASFV genotypes and data on small RNA are needed to substantiate this hypothesis.

The possible role of EVEs in the antiviral response of ticks might also explain why some EVEs have been protected from accumulating mutations. However, it remains unclear why no siRNA or piRNA could be detected for the ASFLI-A104R protein, which is one of the most abundant ASFV proteins in infected wild boar lung cells and infected Vero cells [50], highly conserved between ASFV isolates [51] and whose viral counterpart is crucial for virus replication [51]. While our data might lead to the conclusion that the ASFLI-A104R protein is not translated, the amount of protein might just have been too low for detection or cellular machinery might degrade the protein. Due to the high identity to modern ASFV-A104R sequences, a more recent integration event has also to be considered.

However, further analyses including mass spectrometry analyses, the cloning of the ASFLI-A104R element into a recent ASFV isolate and the biochemical and functional characterisation of the protein should be performed to elucidate a possible function and the nature of ASFLI-A104R.

## Conclusion

In conclusion, we present evidence for endogenous viral elements of the only known DNA arbovirus, ASFV, which might have been integrated into soft tick genomes over 1.46–1.47 million years ago. They serve as templates for siRNA and piRNA thereby possibly protecting the tick against viral infection. We believe that this discovery is an exceptional starting point to study the evolution of ASFV, one of the most dangerous threats to sustainable pig production in Africa, Europe and Asia, as well as the mechanisms by which organisms adapt to the ancient struggle with viral pathogens.

## Methods

### Virus strains

All work with active ASFV was carried out in the FLIs high-security facilities under BSL3+ conditions.

For infection studies, we used an ASFV isolate designated ‘ASFV ken.rie1’. The virus was isolated from *O. porcinus* ticks from Kenya on swine peripheral blood mononuclear cells (PBMCs), passaged twice and titrated on PBMCs as described elsewhere [52]. By sequencing the *p72*, *B602L* and *p54* genes, we compared ‘ASFV ken.rie1’ to known ASFV isolates from GenBank and grouped it into *p72* genotype X, which is known to circulate in East Africa between ticks and indigenous pigs [53]. ‘ASFV-Sardinia’ and ‘ASFV ken06.bus’ (originally received from the European reference laboratory for ASFV (CISA-INIA)) were stored at the German National Reference Laboratory for ASFV as reference material. Both had initially been passaged and titrated on PBMCs.

### Tick rearing, tick infection and tick cell cultures

*O. moubata* ticks, sourced from laboratory colonies maintained at HU-Berlin, Germany, and CIRAD-France as well as *O. porcinus* ticks collected from the field in Kenya and *O. erraticus* ticks collected from the field in Portugal were held at 25 °C and 85% relative humidity in the dark and fed approximately every 2 months on defibrinated pig blood without any additives (heated to 37 °C) through a parafilm membrane without any stimulants using a polystyrene feeding chamber mounted on a heating plate as described elsewhere [54]. Laboratory-reared *O. moubata* from The Spanish National Research Council (CSIC) Salamanca in Spain and The Pirbright Institute in the UK were received and stored in 70% ethanol. DNA from Nigerian *O. savigny* was provided by the University of East London. Tick infection was carried out using third nymphal stage ticks from an ASFV-negative laboratory-reared colony (*O. moubata*) or field ticks (*O. porcinus*). To ensure the absence of ASFV in the field ticks, adult females were fed as described above and all produced eggs were separated. After the death of the adult females, they were tested for ASFV P72-specific DNA by qPCR as described below, and in case of negative results, all offspring were designated ASFV-free, maintained on ASFV free defibrinated pig blood (as described above) and used for infection experiments. For infection, the same feeding system than for colony maintenance [54] was used with ASFV-spiked blood. Ticks of the same species were fed together until repletion and spontaneous detachment. The ticks were manually collected using forceps and held as described above for 28 days. Random ticks were collected on days 0, 7, 14, 21 and 28 post-infection (dpi) and stored at − 80 °C prior to RNA extraction and RT-qPCR analysis as described below. The *O. moubata* cell lines OME/CTVM21, OME/CTVM22, OME/CTVM24, OME/CTVM25, OME/CTVM26 and OME/CTVM27 were maintained in sealed flat-sided culture tubes (Nunc, Thermo Scientific, Waltham, MA) at 28 °C in either L15, L15/H-Lac or L15/MEM medium as described elsewhere [15]. OME/CTVM25 and OME/CTVM26 grow extremely slowly and were therefore only maintained briefly for NGS and qPCR analysis. OME/CTVM21, OME/CTVM22, OME/CTVM24 and OME/CTVM27 were infected with ASFV by adding 0.22 × 10^5^ HAU directly to 2.2-ml cell culture without washing. On days 1, 7, 14, 21 and 28 post-infection, 125 μl cell culture was removed and added to 125 μl PBS and 750 μl TRIzol LS (Thermo Scientific) for RNA extraction. An equal volume of fresh culture medium was added to the culture tubes. All samples were stored at 4 °C until RNA extraction. The medium was changed weekly as described elsewhere [15].

### Nucleic acid extraction

Ticks were homogenised in a 2-ml reaction tube with two 5-mm steel beads and 500 μl PBS using a Tissue Lyser II (Qiagen, Hilden, Germany) for 3 min at 30 Hz. The homogenate was centrifuged at 10,000×*g* for 5 min, and DNA was extracted from the supernatant using the High Pure Template Prep Kit (Roche) according to the manufacturer’s instructions. Tick cell line DNA was extracted using the same kit according to the manufacturer’s instructions.

High-molecular weight DNA was isolated from tick cells using a salting-out protocol [55], slightly modified by adding RNase A (10 mg/ml) for incubation at 37 °C for 60 min in the first step and proteinase K (10 mg/ml) for incubation at 56 °C for another 60 min in the second step.

For RNA extraction, ticks were homogenised in a 2-ml reaction tube with two 5-mm steel beads and 1 ml TRIzol Reagent (Thermo Scientific) using a Tissue Lyser II (Qiagen) for 3 min at 30 Hz. For efficient cell lysis, the homogenate was incubated for 10 min at room temperature. After addition of 200 μl trichlormethane (Sigma Aldrich, St. Louis, MO), samples were thoroughly mixed, incubated for 10 min at room temperature and centrifuged at 10,000×*g* for 10 min at 4 °C. The clear aqueous phase was removed, mixed with 600 μl of 100% ethanol and loaded onto an RNeasy-spin column (Qiagen) for RNA extraction and additional on-column DNA digestion following the manufacturer’s instructions for total RNA extraction. For small RNA extraction, single N3-nymphs were homogenised in 1 ml TRIzol reagent as described above. Small RNA was extracted from the resulting clear aqueous phase using the miRNeasy Mini kit (Qiagen) in combination with the RNeasy MinElute® Cleanup Kit (Qiagen) according to the manufacturer’s protocol.

RNA extraction from tick cell lines was performed using TRIzol LS and RNeasy-spin columns (Qiagen) according to the manufacturer’s protocol. Prior to qRT-PCR analysis, all RNA samples were treated with Turbo-DNase (Life Technologies) for removal of residual DNA.

DNA extraction from museum-stored ticks was done in a DNA cleanroom under ancient-DNA (aDNA) conditions as described elsewhere [56, 57].

### Oligonucleotide design

All primers and probes were designed using the Geneious (v.10.0.9) software suite and are listed in Additional file 19: Table S12 with additional primer sequences obtained from the literature.

### PCR

For PCR reactions, Phusion Green Hot Start II High Fidelity PCR Master Mix (Thermo Scientific) was used according to the manufacturer’s instructions on a C1000 Thermo Cycler (Bio-Rad, Hercules, CA). PCR products were visualised, depending on their length, on 1% or 1.5% agarose gels and visualised by ethidium bromide staining under UV light.

### qPCR

All qPCR reactions were done using the QuantiTect Multiplex PCR NoROX Kit (Qiagen) in a 12.5-μl reaction including 2.5 μl extracted DNA, on a C1000 Thermo Cycler (Bio-Rad) in combination with a CFX96™ Real-Time PCR Detection System (Bio-Rad) according to the manufacturer’s instructions. Primers and probes were premixed (10 pmol primer/μl + 1.25 pmol probe/μl), and 1 μl of the mixture was used per reaction. For ASFV DNA amplification, an OIE-listed qPCR assay was used targeting the viral B646L gene showing a detection limit of 5–10 copies as described elsewhere [58].

Control qPCR systems targeted *O. moubata* mitochondrial (16S rDNA) modified from [59], *O. moubata* chromosomal (enolase) and *O. erraticus* chromosomal (subolesin) housekeeping genes (designed in this study).

### RT-qPCR

All RT-qPCR reactions were performed using the QuantiTect Probe RT-PCR Kit (Qiagen) on a C1000 Thermo Cycler (Bio-Rad) in combination with a CFX96™ Real-Time PCR Detection System (Bio-Rad) according to the manufacturer’s instructions. RNA concentration was measured using the NanoDrop™ 2000 (Thermo Fisher Scientific), and 100 ng RNA in 4 μl (25 ng/μl) was used in a 12.5-μl reaction according to the manufacturer’s protocol. For P72 transcript quantification, the OIE listed qPCR assay [58] was used with the following cycling conditions: 50 °C for 30 min, 95 °C for 15 min, 45 cycles of 95 °C for 1 min, 60 °C for 30 s and 72 °C for 30 s. For all samples tested by RT-qPCR, controls without reverse transcriptase were included to demonstrate the absence of DNA. For absolute quantification, a standard curve was generated, using samples of known copy number produced from cloned gene fragments, and measured by the Quant-iT dsDNA Assay Kit HS (Thermo Fisher Scientific).

### Sanger sequencing

For Sanger sequencing, PCR products were extracted from the agarose gel with the QIAQuick Gel Extraction Kit (Qiagen). The sequencing PCR was done using the BigDye Terminator Kit V1.1 (Thermo Scientific). After purification with NucleoSEQ spin columns (Macherey Nagel, Düren, Germany), samples were sequenced on a Genetic Analyzer 3031XL (Life Technologies, Carlsbad, CA), followed by chromatogram editing with the Geneious (v.10.0.9) software.

### Next-generation sequencing

For IonTorrent PGM sequencing, the libraries lib01543-lib01546, lib01610 and lib01611 were prepared from the six *O. moubata* cell lines as detailed in Additional file 1: Table S1 and sequenced as described elsewhere [60]. The libraries from museum-stored ticks (lib03076-3086) were prepared under aDNA conditions in a DNA cleanroom and sequenced as described elsewhere [56, 57].

The libraries lib02151 and lib02152 were prepared from OME/CTVM21 cells for sequencing on an Illumina MiSeq platform in 300-bp paired-end mode as previously described [61] (Additional file 1: Table S1). The library lib02339 was prepared from OME/CTVM21 cells for sequencing on an Illumina HiSeq platform in 50-bp paired-end mode (Additional file 1: Table S1). Extracted DNA was fragmented to a size of 150 bp (M220 Focused-ultrasonicator™, Covaris, Woburn, MA), and libraries were prepared using NEXTflex™ Dual-Index adapters (Bioo Scientific, Austin, TX) in an automated fashion (SPRIworks Fragment Library System II, Beckman Coulter, Brea, CA). Size exclusion was performed manually aiming at a fragment length peak of 350 bp (Agencourt® AMPure® XP Beads, Beckman Coulter). The library was then amplified (AccuPrime™ Taq DNA Polymerase, high fidelity, Invitrogen, Carlsbad, CA), purified (Agencourt® AMPure® XP Beads) and quality-checked (High Sensitivity DNA Kit, Agilent Technologies). The final library was quantified (KAPA Library Quantification Kit, Roche) and sequenced (Illumina) according to the manufacturer’s instructions.

For MinION sequencing, high-molecular weight tick cell DNA was fragmented using G-Tubes (Covaris), followed by library preparation (libMinION_1) using the 1D^2^ Sequencing Kit (R9.5) (Oxford Nanopore, Oxford, UK) according to the manufacturer’s protocol. Sequencing was performed on the MinION MIN-101B system (Oxford Nanopore).

For small RNA sequencing, library preparation from enriched small RNA samples was performed using the Ion Total RNA-Seq Kit v2 (Thermo Scientific). The libraries lib02965 (mRNA), lib02966, lib02967, lib03460 and lib03506 (small RNA) were prepared for, and sequenced on, an IonTorrent S5 (Thermo Fisher Scientific) as described elsewhere [62].

### Amplicon sequencing for assembly validation

For the validation of the two ASFLI element containing contigs (4.3 and 6.8 kb), PCR reactions were performed as described above. PCR products were extracted from the agarose gel with the QIAQuick Gel Extraction Kit (Qiagen) and sequenced (lib02035) on an Illumina MiSeq platform in 300-bp paired-end mode as previously described [61]. The data was analysed by assembly using Newbler 3.0 and alignment with the originally assembled sequences using MAFFT v7.388 in Geneious.

### Data analysis

The metagenomic classification software pipeline RIEMS [63] was used on lib01543-01546 and lib01610-01611 (Additional file 1: Table S1). For further data analysis and proof of integration of ASFV-like sequences into the tick cell genome, overlapping read pairs in the MiSeq data sets (lib02151 and lib02152) were merged to single reads using FLASH v1.2.11 [64]. Subsequently, the merged reads were assembled together with data from lib02339 and libMinION_1 using SPAdes v3.11.1 [16] in the mode of read error correction prior to assembly. In total, 66,745 contigs were assembled with standard assembly parameters and automatically determined *K*-mer length of 21, 33, 55, 77, 99 and 127. The resulting contigs were blasted (BLASTn, NCBI, v2.6.0+) against a customised database comprising all sequences with the NCBI taxonomy ID 10497 (African swine fever virus) (as of 16 January 2018). Hits were filtered using a cut-off *e* value of 1 × 10^−4^ and a minimum alignment length of 150 bp, resulting in 34 contigs. These were then blasted against the complete NCBI database (the non-redundant nucleotide collection) to reliably identify and annotate ASFV-like sequences and areas of the host genome using default parameters. BLASTp search of > 500 bp ORFs was performed against the ‘non-redundant protein sequences’ database using default parameters (Additional file 20: Figure S7).

ASFLI-element sequences from mRNA sequencing data and additional *O. moubata* transcriptome data [18] downloaded from GenBank were identified by mapping using the Newbler software 3.0 (Roche) against a database containing all known ASFV sequences and previously obtained ASFLI element sequences.

DNA sequence data from lib03101 and lib03102 (*O. porcinus* ticks) were assembled using SPAdes v3.11.1 [16] in the mode of ‘read error correction prior to assembly’ with standard assembly parameters and automatically determined *K*-mer length of 21, 33, 55, 77, 99 and 127. The resulting contigs were blasted against a customised database comprising all sequences with the NCBI taxonomy ID 10497 (African swine fever virus) (as of 10 May 2019). Obtained hits were filtered as described above (Additional file 20: Figure S7).

DNA sequence data from the museum ticks (lib03076-03086 and lib03376-03377) were mapped against a database comprising all available ASFLI-element sequences either from the *O. moubata* cell lines or from *O. porcinus* ticks using Bowtie (v.1.1.2) with default parameters. Mapped reads were assembled using SPAdes v3.11.1. Small RNA data were used with and without deduplication using dedupe from the BBTools package [65]. Reads were mapped against ASFV genomes or ASFLI-element-containing databases using Bowtie (1.1.2) in Geneious with 22-nt seed length for siRNA and 28-nt seed length for piRNA and one allowed mismatch. The piRNA ‘ping-pong signature’ was analysed using PingPongPro v1.0 [66] in default mode with the –b option (creates additional browser track files, which are suitable for display in common genome browsers), and results were analysed in Geneious.

For the generation of full-length tick mitochondrial genomes, sequence data was mapped against a database containing all available *Ornithodoros* full-length mitochondrial sequences (as of 20 June 2019) using Newbler 3.0 and Bowtie 2 (2.3.0), and mapped reads were assembled using Newbler 3.0 and SPAdes v.3.11.1 with default parameters and automatically chosen *K*-mer length of 21, 33 and 55.

### Phylogenetic analysis

For the identification of homologous genes from other viral genera, BLASTp (protein-protein BLAST) was used with amino acid sequences from the translated ASFV Ken06/Bus F1055L (helicase) and EP1242L (RNA polymerase subunit 2) genes using the non-redundant protein sequences database. All viral sequences identified by BLASTp, covering at least 60% of the query, were used for further analysis. This yielded sequences from the NCLDV, including Faustoviruses, Pacmanvirus, Kaumoebavirus and Marseillevirus as well as African swine fever virus. These sequences together with the corresponding translation of the ASFLI-elements were aligned using MUSCLE in MEGA [67, 68]. Nucleotide sequences of the EP1242L gene and equivalents from ASFV, Faustovirus, Pacmanvirus and Kaumoebavirus were also downloaded from GenBank. These nucleotide sequences together with the ASFLI-element were again aligned using MUSCLE in MEGA. A maximum likelihood tree was calculated from an alignment of NCLDV, ASFV and ASFV-like sequences in MEGA. The Jones-Taylor-Thornton (JTT) model allowing gamma-distributed rates between sites (four categories), considering sites with > 95% data, and 100 bootstraps was used [69]. More details about the methods used for the phylogenetic analyses are presented in Additional file 5: Supplementary Appendix.

Tick mitochondrial genomes were aligned using MAFFT v7.388 in Geneious. The phylogenetic tree was constructed using IQ-TREE v1.6.5 with standard model selection, resulting in the best-fit model TIM2+F+R3 (AC=AT, CG=GT and unequal base frequencies + empirical base frequencies + FreeRate model with 3 categories).

### Clock rate estimates and Bayesian time-scaled trees

To obtain an initial clock rate estimate, the ASFV nucleotide sequences were analysed using BEAST 1.8.4 [70, 71]. Several different clock models were employed, and results were evaluated with the marginal likelihood estimation using path sampling and the stepping-stone sampling feature within BEAST [72]. The clock models were: ‘strict’, ‘uncorrelated relaxed log-normal’, ‘uncorrelated relaxed gamma’, ‘uncorrelated relaxed exponential’, ‘random local clock’ and ‘fixed local clock’ [73–75]. Apart from the clock models, the substitution model TN93 with gamma-distributed site-to-site rate variation over four categories and constant population size tree prior model (effective population size) were used. Each BEAST run used a Markov chain Monte Carlo (MCMC) chain with a length of 10,000,000 steps, sampling every 1000 steps and discarding the first 10% as burn-in.

All estimations of the time to the most recent common ancestor (TMRCA) are given in millions of years ago (mya). More details about the methods used for the molecular clock analyses are presented in Additional file 5: Supplementary Appendix.

### Protein expression and purification in *E. coli* and rabbit immunisation

The reconstructed ASFLI-A104R gene was custom-synthesised and inserted into vector pMA-T (Geneart, Invitrogen). It was then amplified by PCR, and the products were inserted into vector pMAL-p2X (New England Biolabs, Ipswich, MA). After transformation of *E. coli* (TB1) with the plasmid and induction with isopropyl-*β*-d-thiogalactopyranosid (IPTG), the maltose-binding protein (MBP) fusion protein was purified by affinity solid-phase extraction on an amylose resin as suggested by the manufacturer (New England Biolabs). Rabbits were immunised by intramuscular injections of 400 μg purified MBP–A104R fusion protein every 4 weeks over a period of 4 months. Blood was taken monthly and stored at 4 °C overnight after collection, followed by centrifugation for 10 min at 453×*g* at room temperature. The resulting sera were stored at − 20 °C.

### Transfection

Approximately 10^6^ HEK 293T (ATCC® Number: CRL-11268™) cells were cultivated for 24 h in 6-well plates. Using 5 μg of polyethylenimine (PEI) [76], the cells were transfected with 2.5 μg plasmid pCAGGS-Zecke-A104R in which transgene expression is directed by a hybrid HCMV major immediate-early enhancer/chicken-actin promoter [77]. The plasmid/PEI mix was added to the cell culture after pre-incubation at room temperature for 20 min and centrifuged at 600×*g* for 60 min at 26 °C to enhance transfection efficacy. After 5 h of incubation at 37 °C, the medium was replaced by fresh medium, and after 48 h of further incubation, cells were harvested and lysed in 500 μl sample buffer for SDS-PAGE.

### SDS-PAGE and immunoblotting

SDS-PAGE was performed with a Mini-Protean Tetra System (Bio-Rad) using hand-cast SDS gels with 15% polyacrylamide according to Laemmli [78]. After separation, proteins were blotted onto nitrocellulose membranes (Whatman, Maidstone, UK) using a Trans-Blot SD Semi-Dry Transfer Cell (Bio-Rad) under conditions suggested by the manufacturer. Membranes were blocked by incubation in PBS supplemented with 6% milk powder (Sigma Aldrich) and 10% horse serum (Sigma Aldrich) overnight at 4 °C. After consecutive washes with 0.3% and 0.1% Tween20 in PBS, the blot was incubated with a 1:2500 dilution of the rabbit serum (collected following the third immunisation), washed again with PBS-Tween20 as described above, and finally incubated with a 1:20,000 dilution of a peroxidase-conjugated secondary goat anti-rabbit antibody (Dianova, Hamburg, Germany). Signals were visualised using the ECL kit (Clarity Western ECL, Bio-Rad, Hercules, CA) as suggested by the manufacturer.

### Statistical analysis

To calculate the number of ticks needed for the infection experiment, we conducted sample size calculation using R-studio (https://www.rstudio.com) as described elsewhere [79] with an assumed prevalence of 0.9 in one population and 0.3 in the second population. In order to obtain a power of 80% (beta = 0.2) and a confidence level of 99% (alpha = 0.01), the required sample size was calculated to be ten animals per group.

## Supplementary information

**Additional file 1: Table S1.** Overview of NGS experiments conducted in this study and data obtained from the literature. *Ornithodoros moubata* cell line names have been abbreviated by removal of “/CTVM” and *Ornithodoros porcinus* and *Ornithodoros moubata* have been abbreviated for convenience.

**Additional file 2: Table S2.** ASFV-like transcripts detected in data from six *Ornithodoros moubata* cell lines and *O.moubata* ticks by mRNA-sequencing and data analysis from published sequences ASFV-like sequences from tick cell libraries lib01543-46 und lib01610-11 (total RNA) were identified using RIEMS. The resulting reads were blasted against the NCBI BLASTn database for confirmation and annotation. *Ornithodoros moubata* transcriptome data from a previous study^42^ was downloaded from GenBank, and ASFV-like sequences were identified by mapping the data against a tailored database containing all known ASFV sequences and all previously obtained ASFV-like sequences. The resulting reads and contigs were annotated after a BLASTn search against the entire BLASTn database. Sequencing data from the mRNA enriched OME21 library lib02965 was mapped against a tailored database containing all known ASFV sequences and all previously obtained ASFV-like sequences, and resulting contigs were blasted against the entire BLASTn database. Per sequence, only the hit with the lowest e-value is shown.

**Additional file 3: Table S3.** BLASTn results of SPAdes-assembled contigs containing ASFLI- and other endogenous viral elements detected in the OME/CTVM21 genome. Shown are hits with the lowest e-value for viral, tick and mobile genetic element-related genes, as obtained by Blastn against the entire NCBI database.

**Additional file 4: Table S4.** BLASTp results of all ORFs >500 bp on SPAdes-assembled contigs containing ASFLI- and other endogenous viral elements detected in the OME/CTVM21 genome Shown are hits with the lowest e-value for viral-, tick and mobile genetic element-related proteins, as obtained by Blastp of ORFs >500 bp against the entire NCBI database.

**Additional file 5: Supplementary Appendix.** Phylogenetic and molecular clock analyses of ASFLI-elements from different tick genomes and ASFV using different clock-rates and substitution models implemented in BEAST. **Supplementary Appendix. Figure A1-A5, Tables A1-A7**. FigA1- Phylogenetic tree of ASFV and ASFLI-elements. FigA2 – Phylogenetic tree of NCLDV including ASFV and ASFLI-elements. FigA3 – Time-scaled tree for partial EP1242L sequences from tick samples and reference ASFV (non-integrated). FigA4 - Time-scaled tree for partial EP1242L sequences from tick samples only (5e-7 substitutions per site per year). FigA5 - Time-scaled tree for partial EP1242L sequences from tick samples (1e-8 substitutions per site and year). Table A1 - Summary of EP1242L fragments derived from ticks and the tick cell line OME/CTVM21 (OME21) used in the analysis. Table A2 - Indels in the tick sample sequences relative to the start of EP1242L in ASFV|KM111295|Kenya|Ken06/Bus|2006. Table A3 - Estimated root height and overall mean clock rate for strict clocks with fixed priors. Table A4 - Estimated root height and overall (averaged over all branches) mean clock rate for relaxed clocks with fixed priors of the rate of the relaxed clock (some variation). Table A5 - Estimated root height and overall (averaged over all branches) mean clock rate for relaxed clocks with normal or log-normal priors on the rate of the relaxed clock (most variation). Table A6 - Estimated root height and overall (averaged over all branches) mean clock rate for strict clocks with log-normal priors on the rate of the strict clock for the five ASFV-like sequences from tick samples only. Table A7 - Marginal likelihood estimation using Path Sampling and Stepping Stone Sampling, showing that the log-normal clock rate prior with mean = 5e-7 is best, but not significantly better than the other clock rates.

**Additional file 6: Table S5.** Results of tick and tick cell line screening for ASFLI-elements by qPCR. DNA from single ticks and every tick cell line (*n*=1) was extracted and analysed by qPCR for six ASFV-like genes as described in the ‘Material and methods’ section. All samples were tested with a qPCR control targeting a tick housekeeping gene to demonstrate successful DNA extraction and presence of tick DNA. Absence of ASFV in samples producing false positive results was proven by an OIE-listed qPCR.

**Additional file 7: Table S6.** BLASTn results of SPAdes-assembled contigs from O*rnithodoro porcinus* France and Kenya17 containing ASFLI-elements. Shown are hits with the lowest e-value for ASFV-genes as obtained by Blastn against the entire NCBI database.

**Additional file 8: Figure S1.**Similarity between ASFLI-element contigs identified in *Ornithodoros moubata* cells and *Ornithodoros porcinus* ticks. Contigs were assembled using SPAdes 3.13. on sequencing data obtained from *O. porcinus* ticks (OPF) and and OME/CTVM21 cells (OME) using default parameters. After contig identification by BLASTn and BLASTp search against the entire NCBI database and annotation, contigs were aligned using MAFFT v7.388 in Geneious. To analyse the possible existence of multiple copies of the piggyback transposable element, coverage was calculated in Geneious from mapping of libs 02151-2 and lib02339 against Contig 22316_OME and lib03101-2 against Contig 955_OPF using Bowtie2 with default paramteres in the “very sensitive” mode.

**Additional file 9: Table S7.** SPAdes-assembled contigs from deep sequencing data from libraries AGL001 and MPA001, as generated from museum-stored ticks. Data from libraries from museum-stored tick were mapped against ASFLI-element-containing databases of *Ornithoidoros moubata* (AGL001) and *Ornithodoros porcinus* (MPA001) using Bowtie2 (v.2.3.4.3) with default parameters. Subsequently, mapped reads were assembled using SPAdes and aligned to the corresponding ASFLI- element-contigs using MAFFT (v. 7.388) in Geneious.

**Additional file 10: Table S8.** qRT-PCR results of *Ornithodoros* ticks experimentally infected with different ASFV-genotype isolate. Shown are ASFV-P72 transcript-specific Cq-values of third nymphal stage ticks fed with defibrinated pig blood, containing either 1 x 10^4^ HAU/ml or 1 x 10^6^ HAU/ml ASFV-ken.rie1 (GT X) (A-D), 1 x 10^5^ HAU/ml ASFV-Ken06.bus (GT IX) (E-F) or 1 x 10^4^ HAU/ml ASFV-Sardinia (GT I) (G-H). Due to the limited number of field ticks available and feeding under artificial conditions, fifteen *Ornithodoros porcinus* ticks were collected in each of three experiments and ten in two experiments while for the laboratory-reared *Ornithodoros moubata*, twenty-five individuals were collected in each of three experiments and fifteen in one experiment. All samples were stored at – 80 °C until RNA-extraction and ASFV transcript-specific qRT-PCR analysis as described in the ‘Material and methods’ section.

**Additional file 11: Figure S2.** Read length distribution and piRNA nucleotide bias of small RNA libraries. The length distribution is shown from small RNA libraries before and after removal of duplicate reads. Blue colored bars indicate siRNA (22nt) and piRNA (28-29nt) fractions **(A)**. Reads of the piRNA fraction (28-29nt) were extracted from raw and deduplicated libraries, trimmed at the 3’ end to 28 nt for analysis and nucleotide frequencies for every position were calculated using R-studio (https://www.rstudio.com) for the entire dataset **(B)** and for non-deduplicated reads mapped to all ASFLI-elements and all available ASFV whole-genome sequences using Bowtie in Geneious with 28 nt seed length and one allowed missmatch **(C)**. piRNA from *O. porcinus* did only map to the ASFV forward strand.

**Additional file 12: Figure S3.** siRNA/piRNA mapping against ASFV-R8 (G IX) and two ASFLI-elements. siRNA (22 nt) and piRNA (29 nt) fractions from *Ornithodoros moubata* were individually mapped against available ASFV whole-genome sequences (the one showing the most mapped reads, ASFV-R8 (GT IX) is shown) and two exemplary *O. moubata* ASFLI-element-containing contigs (NODE5010 and NODE28076) using Bowtie (1.1.2) in Geneious. Marked (arrows) are the most abundant small RNA molecules related to the ASFV genes and ASFLI-elements.

**Additional file 13: Figure S4.** siRNA/piRNA mapping against ASFV-Kenya1950 (GT X), Warthog (G IV) and two ASFLI-elements. siRNA (22 nt) and piRNA (28-29 nt) fractions from *Ornithodoros porcinus* were individually mapped against available ASFV whole-genome sequences (the ones showing the most mapped reads, ASFV-Kenya1950 (GT X), ASFV Wathog (GT IV) are shown) and two exemplary *O. porcinus* ASFLI-element containing contigs (NODE955 and NODE1089) using Bowtie 1 (1.1.2) in Geneious. Marked (arrows) are the most abundant small RNA molecules relating to the ASFV genes and ASFLI-elements.

**Additional file 14: Figure S5.** siRNA/piRNA mapping against ASFV and ASFLI-element database. siRNA (22 nt) and piRNA (28-29 nt) fractions from *Ornithodoros porcinus* and *Ornithodoros moubata*, before and after deduplication, were individually mapped against ASFV whole-genome sequence and the *O. moubata* or *O. porcinus* ASFLI-element containing datasets using Bowtie 2 (2.3.0) in Geneious.

**Additional file 15: Table S9.** Results of small RNA sequencing and mapping against ASFV and ASFLI-elements. Small RNA was sequenced from *Ornithodoros porcinus* and *Ornithodoros moubata* nymphal stage ticks. After deduplication using BBMap, 22 nt siRNA and 28-29 nt piRNA fractions were extracted and mapped against ASFV and ASFLI-elements using Bowtie.

**Additional file 16: Table S10.** *O. moubata* piRNA ping-pong signature analysed by PingPongPro v1.0 with default parameters and -b option (creates additional browser track files, which are suitable for display in common genome browsers). The closer the Score-value (1 minus the FDR-value: estimated fraction of signatures that have the same combination of properties, but that are not true ping-pong signatures) which is calculated from adenine bias at pos. 10 of the piRNA, stack height - e.g. the number of reads that make up the stack and independence of local coverage is to 1, the more likely is a true ping-pong signature.

**Additional file 17: Table S11.** *O. porcinus* piRNA ping-pong signature analysed by PingPongPro v1.0 with default parameters and -b option (creates additional browser track files, which are suitable for display in common genome browsers). The closer the Score-value (1 minus the FDR-value: estimated fraction of signatures that have the same combination of properties, but that are not true ping-pong signatures) which is calculated from adenine bias at pos. 10 of the piRNA, stack height - e.g. the number of reads that make up the stack and independence of local coverage is to 1, the more likely is a true ping-pong signature.

**Additional file 18: Figure S6.** The reconstructed ASFV-like A104R protein is highly similar to its ASFV homologue **(A)** A rabbit antiserum raised against the reconstructed A104 gene recognised a flag-tagged and an untagged version of A104R protein (lanes A104-Flag and A104, respectively), but showed no specific reaction with extracts of tick cell lines OME/CTVM21, OME/CTVM22, OME/CTVM24, and OME/CTVM27. In extracts of WSL-HP cells infected with ASFV Kenya 1033, the serum reacted with a single band of 12 kDa which is similar to the calculated molecular weight of ASFV A104R (11.6 kDa). **(B)** The Coomassie stained gel confirms equal loading with proteins.

**Additional file 19: Table S12.**Primer and probe sequences used in this study.

**Additional file 20: Figure S7.** BLAST-analysis for identification of ASFLI-element containing contigs and annotation**.** 66,745 SPAdes assembled contigs were blasted (BLASTn, NCBI, v2.6.0+) against a customised database comprising all sequences with the NCBI taxonomy ID 10497 (African swine fever virus) (as of 16 January 2018). Hits were filtered using a cut off e-value of 1x10^-4^ and a minimum alignment length of 150 bp, resulting in 34 contigs. These were then blasted against the complete NCBI database (The non-redundant nucleotide collection) to reliably identify and annotate ASFV-like sequences and areas of the host genome using default parameters. BLASTp search of >500 bp ORFs was performed against the “Non-redundant protein sequences” database using default parameters.

## Data Availability

The datasets supporting the conclusions of this article are available from the International Nucleotide Sequence Database Collaboration (INSDC) under study accession number PRJEB26739, https://www.ebi.ac.uk/ena/data/search?query=PRJEB26739 [80].
