# Supplementary material for: Identification of African swine fever virus-like elements in the soft tick genome provides insights into the virus’ evolution
Source: BMC Biol. 2020 Oct 8;18:136. doi: 10.1186/s12915-020-00865-6 (PMC7542975; doi:10.1186/s12915-020-00865-6)
Supplement: Supplementary file 5 — Additional file 5: Supplementary Appendix. Phylogenetic and molecular clock analyses of ASFLI-elements from different tick genomes and ASFV using different clock-rates and substitution models implemented in BEAST. Supplementary Appendix. Figure A1-A5, Tables A1-A7. FigA1- Phylogenetic tree of ASFV and ASFLI-elements. FigA2 – Phylogenetic tree of NCLDV including ASFV and ASFLI-elements. FigA3 – Time-scaled tree for partial EP1242L sequences from tick samples and reference ASFV (non-integrated). FigA4 - Time-scaled tree for partial EP1242L sequences from tick samples only (5e-7 substitutions per site per year). FigA5 - Time-scaled tree for partial EP1242L sequences from tick samples (1e-8 substitutions per site and year). Table A1 - Summary of EP1242L fragments derived from ticks and the tick cell line OME/CTVM21 (OME21) used in the analysis. Table A2 - Indels in the tick sample sequences relative to the start of EP1242L in ASFV|KM111295|Kenya|Ken06/Bus|2006. Table A3 - Estimated root height and overall mean clock rate for strict clocks with fixed priors. Table A4 - Estimated root height and overall (averaged over all branches) mean clock rate for relaxed clocks with fixed priors of the rate of the relaxed clock (some variation). Table A5 - Estimated root height and overall (averaged over all branches) mean clock rate for relaxed clocks with normal or log-normal priors on the rate of the relaxed clock (most variation). Table A6 - Estimated root height and overall (averaged over all branches) mean clock rate for strict clocks with log-normal priors on the rate of the strict clock for the five ASFV-like sequences from tick samples only. Table A7 - Marginal likelihood estimation using Path Sampling and Stepping Stone Sampling, showing that the log-normal clock rate prior with mean = 5e-7 is best, but not significantly better than the other clock rates. [file 12915_2020_865_MOESM5_ESM.docx]

## **Supplementary Appendix 1 Phylogenetic analysis and molecular clock analysis**

## **Sequence fragments**

ASFV-like sequences of the gene EP1242L were found in the samples from four *Ornithodoros* spp. ticks originating from Angola, Tanzania (previously known as German East Africa), Kenya and the *Ornithodoros moubata* cell line OME/CTVM21 (grouped hereafter as “tick samples”), along with fragments of other genes (different fragments in different samples). The entire EP1242L viral gene was not found; however fragments of over 1500bp in length were recovered from each of the samples. EP1242L codes for the DNA-directed RNA polymerase subunit beta in ASFV.

The ASFV-like fragments were aligned to the EP1242L of a reference ASFV genome - ASFV|KM111295|Kenya|Ken06/Bus|2006, and the first 1751 nucleotides in the alignment were used (Table A1). The sequences contained a number of deletions and one insertion as compared to Ken06/Bus.

| **Sample number** | **Sample Name or reference virus** | **Fragment Length** | **Length used** |
| --- | --- | --- | --- |
| *(reference)* | *ASFV\|KM111295\|Kenya\|Ken06/Bus\|2006* | *3729* | *1751* |
| 1 | Omoubata\|Original_Contig\|2017 | 2562 | 1641 |
| 2 | AGL001\|Angola\|1900 | 2561 | 1641 |
| 3 | OME21\|tickcells\|2018 | 2562 | 1641 |
| 4 | MPA001A\|German-East-Africa\|1906 | 1630 | 1630 |
| 5 | Oporcinus\|Oporc955_FLI\|2019 (Kenya) | 1638 | 1638 |

Table A1: **Summary of EP1242L fragments derived from ticks and the tick cell line OME/CTVM21 (OME21) used in the analysis**

| **Start** | **End** | **Length** | **Ref** | **1** | **2** | **3** | **4** | **5** | **Include** |
| --- | --- | --- | --- | --- | --- | --- | --- | --- | --- |
| 9 | 15 | 7 | NN | NN | NN | NN | D1 | D1 | Yes |
| 255 | 270 | 16 | NN | D1 | D1 | D1 | NN | NN | Yes |
| 407 | 412 | 6 | NN | D1 | D1 | D1 | NN | NN | Yes |
| 484 | 484 | 1 | NN | NN | NN | NN | D1 | D1 | Yes |
| 524 | 532 | 9 | NN | D1 | D1 | D1 | D1 | D1 |  |
| 563 | 581 | 19 | NN | D1 | D1 | D1 | D1 | D1 |  |
| 633 | 648 | 16 | NN | D2 | D2 | D2 | D1 | D1 | Yes |
| 668 | 675 | 8 | NN | D1 | D1 | D1 | D1 | D1 |  |
| 742 | 750 | 9 | NN | NN | NN | NN | D1 | D1 | Yes |
| 818 | 822 | 5 | NN | NN | NN | NN | D1 | D1 | Yes |
| 961 | 969 | 9 | NN | NN | NN | NN | D1 | D1 | Yes |
| 1013 | 1023 | 11 | NN | NN | NN | NN | D1 | D1 | Yes |
| 1096 | 1105 | 10 | NN | D1 | D1 | D1 | D1 | D1 |  |
| 1385 | 1393 | 9 | NN | D1 | D1 | D1 | D1 | D1 |  |
| 1440 | i |  | NN | II | II | II | NN | NN | Yes |
| 1456 | 1457 | 2 | NN | NN | NN | NN | D1 | D1 | Yes |
| 1677 | 1683 | 7 | NN | D1 | D1 | D1 | NN | NN | Yes |
| 1730 | 1737 | 8 | NN | D1 | D1 | D1 | D1 | NN | Yes |
| 1747 | 1747 | 1 | NN | NN | NN | NN | D1 | NN | Yes |
| 1751 | 1751 | 1 | NN | NN | NN | NN | NN | D1 | Yes |

Table A2: **Indels in the tick sample sequences relative to the start of EP1242L in ASFV|KM111295|Kenya|Ken06/Bus|2006.** NN = nucleotide sequence, D1 = deletion region 1, D2 = deletion region 2, II = insertion region 2. The include columns displays sites included in the time scale estimation (Page 9 of this Appendix).

## **Maximum Likelihood Trees and Relation to ASFV**

### **Phylogeny from nucleotide sequences**

A maximum likelihood tree of the aligned nucleotide sequences of the samples, together with 17 publicly available ASFV EP1242L sequences (non-integrated) was created using MEGA7 with the Tamura-Nei model, gamma-distributed site-to-site rate variation (four categories), and 100 bootstraps.


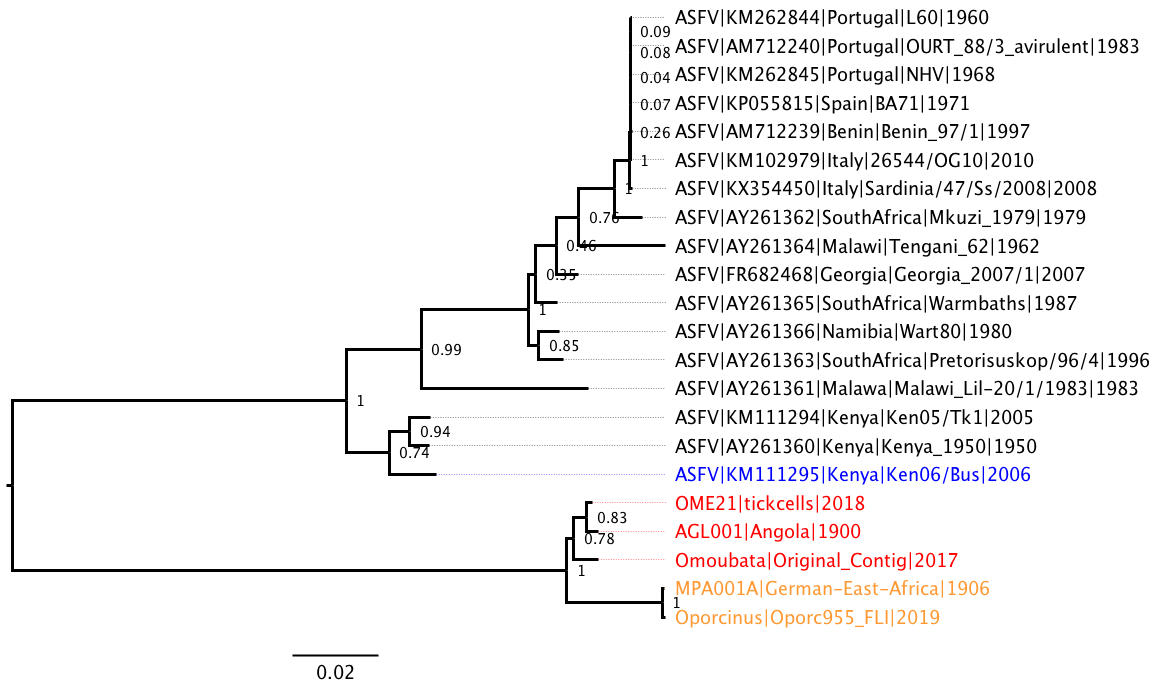


Figure A1: **Phylogenetic tree of ASFV and ASFLI-elements.** Maximum likelihood tree of the ASFV-like nucleotide fragments from the tick samples and of partial ASFV EP1242L genes (non-integrated)

The tree in Figure A1 shows that the viral gene fragments in the samples from ticks are related to ASFV but appear as outgroups. This is to be expected since the viral gene fragments were integrated into the tick genomes and evolved with the host species, whereas the sequences from free ASFV have been evolving independently in the viral population.

### **Phylogeny from amino acid sequences**

To further show the relationship between the ASFV-like sequence fragments and other viruses, a maximum likelihood tree including EP1242L-equivalent sequences from other, more distantly related Megavirales was inferred in MEGA7 from amino acid sequences using the JTT (Jones-Taylor-Thornton) model, gamma-distributed site-to-site rate variation in four categories and 100 bootstraps.


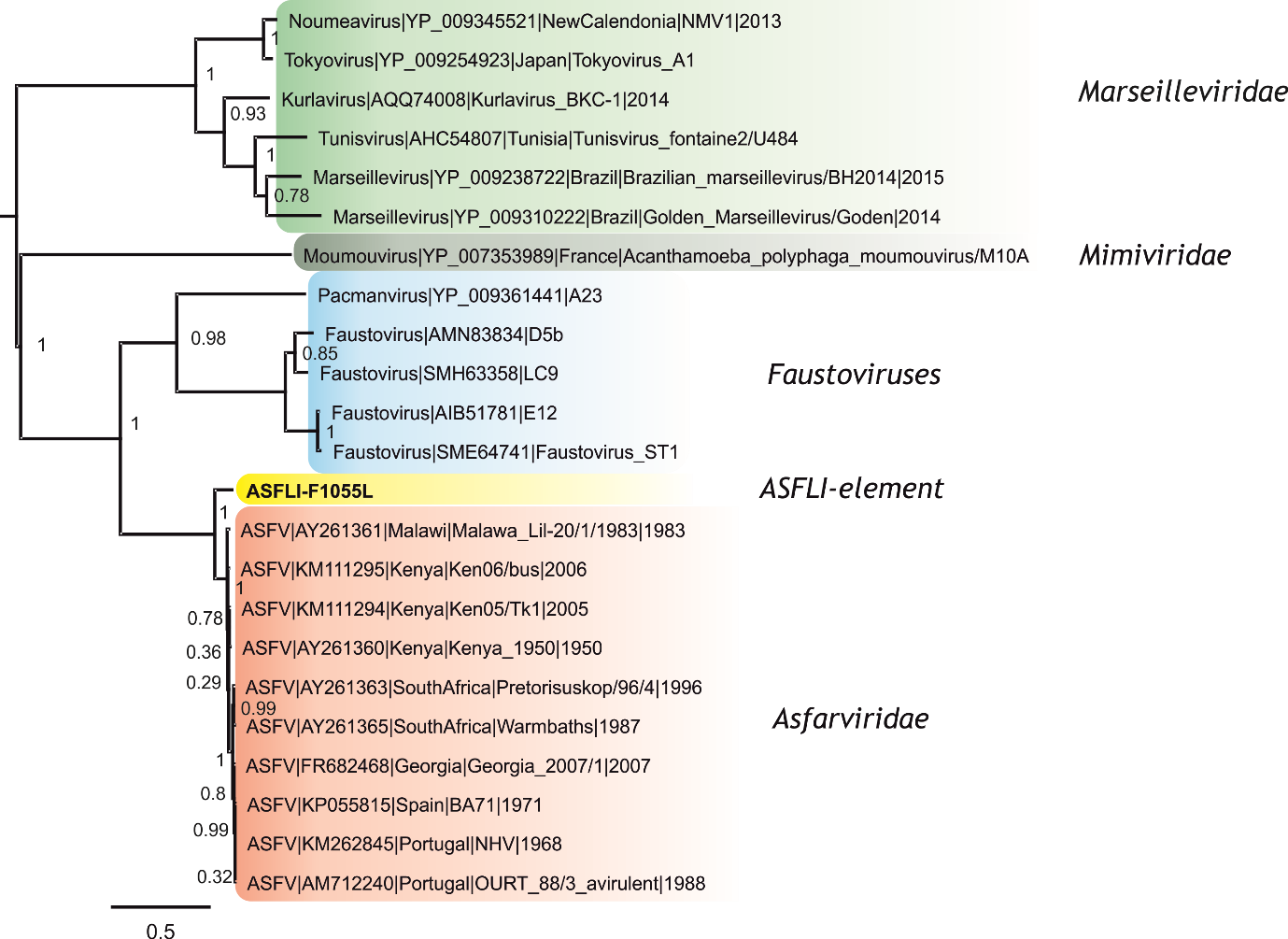


Figure A2: **Phylogenetic tree of NCLDV including ASFV and ASFLI-elements.** Maximum likelihood tree of the ASFV-like amino acid sequences from the tick samples, and of partial ASFV F1055L sequences of (non-integrated) and NCLDV members.

## **Time-scale estimation**

### **Initial estimation**

To estimate the time-scale for divergence of the tick samples and ASFV (non-integrated), time-scaled phylogenies were inferred using BEAST 1.10.4 with the following model settings: a Tamura-Nei 93 (TN93) nucleotide substitution model with gamma-distributed site-to-site rate variation in four categories, a strict or uncorrelated relaxed lognormal clock and a constant population size. Initially, the EP1252L sequence fragments from the five ticks together with the Ken06/Bus sequence reference were used. Several priors for the overall clock rate were compared, drawn from a normal or log-normal distribution with means and standard deviations of between 1e-8 substitutions per site and year to 5e-6 per site and year. The set of values for the clock rate priors were chosen to reflect typical DNA molecular clock rates (lower rates for host, higher rates for ASFV).


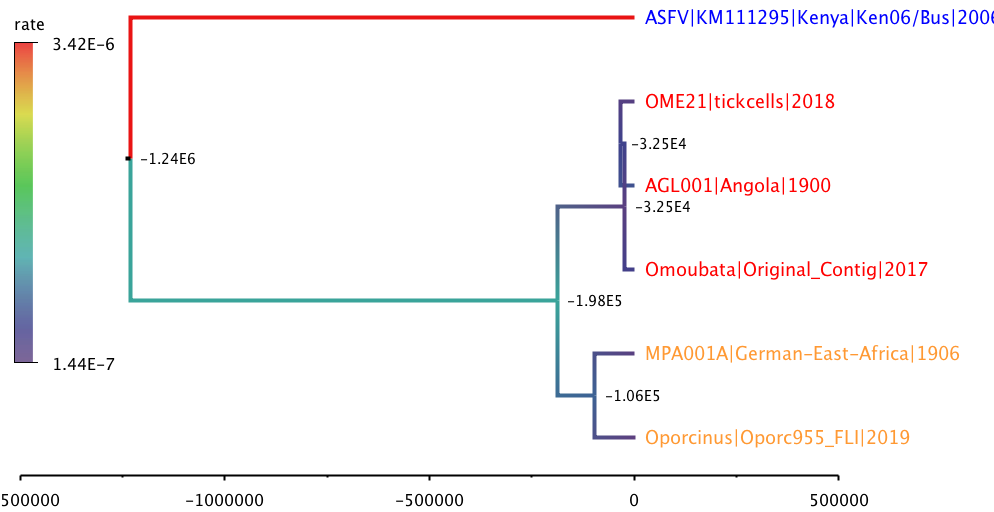


Figure A3: **Time-scaled tree for partial EP1242L sequences from tick samples and reference ASFV (non-integrated).** The tree was inferred using an uncorrelated relaxed log-normal molecular clock and log-normal clock rate prior with mean and standard deviation of 5e-6 substitutions per site and year. Branches are coloured according to the inferred molecular clock rate, dark blue = 1.4e-7 (slow) to red = 3.4e-6 (fast) substitutions/site x year. Node ages are indicated in years BCE.

|  |  | **Root Height** | **Root Age statistics** | | | | | **Mean Rate** |
| --- | --- | --- | --- | --- | --- | --- | --- | --- |
| **Clock** | **Prior** | **mean** | **mean** | **median** | **95% Lower** | **95% Upper** | **ESS** | **mean** |
| Strict | 1e-6 fix | 9.75E+04 | -9.55E+04 | -9.52E+04 | -8.45E+04 | -1.07E+05 | 8389 | 1.00E-06 |
| Strict | 1e-7 fix | 9.76E+05 | -9.74E+05 | -9.71E+05 | -8.69E+05 | -1.09E+06 | 8203 | 1.00E-07 |
| Strict | 1e-8 fix | 9.74E+06 | -9.74E+06 | -9.71E+06 | -8.66E+06 | -1.09E+07 | 8397 | 1.00E-08 |

Table A3: **Estimated root height and overall mean clock rate for strict clocks with fixed priors**

|  |  | **Root Height** | **Root Age statistics** | | | | | **Mean Rate** |
| --- | --- | --- | --- | --- | --- | --- | --- | --- |
| **Clock** | **Prior** | **mean** | **mean** | **median** | **95% Lower** | **95% Upper** | **ESS** | **mean** |
| RelaxLn | 5e-6 fix | 3.02E+06 | -3.01E+06 | -9.10E+05 | -4.04E+03 | -1.17E+07 | 71 | 7.86E-07 |
| RelaxLn | 1e-6 fix | 9.17E+07 | -9.17E+07 | -1.98E+07 | -2.82E+04 | -3.88E+08 | 112 | 4.94E-08 |
| RelaxLn | 5e-7 fix | 1.07E+09 | -1.07E+09 | -1.64E+08 | -5.91E+04 | -5.16E+09 | 22 | 3.49E-08 |
| RelaxLn | 1e-7 fix | 3.54E+09 | -3.54E+09 | -6.48E+08 | -4.11E+05 | -1.46E+10 | 38 | 3.12E-09 |
| RelaxLn | 5e-8 fix | 2.02E+09 | -2.02E+09 | -1.47E+09 | -4.57E+06 | -5.24E+09 | 100 | 2.48E-10 |
| RelaxLn | 1e-8 fix | 7.79E+12 | -7.79E+12 | -2.16E+12 | -7.30E+08 | -3.51E+13 | 60 | 7.07E-13 |

Table A4: **Estimated root height and overall (averaged over all branches) mean clock rate for relaxed clocks with fixed priors of the rate of the relaxed clock (some variation)**

|  |  | **Root Height** | **Root Age statistics** | | | | | **Mean Rate** |
| --- | --- | --- | --- | --- | --- | --- | --- | --- |
| **Clock** | **Prior** | **mean** | **mean** | **median** | **95% Lower** | **95% Upper** | **ESS** | **mean** |
| RelaxLn | 5e-6 log | 7.94E+06 | -7.94E+06 | -1.23E+06 | -3.34E+07 | 5.23E+02 | 107 | 7.76E-07 |
| RelaxLn | 5e-6 nor | 2.32E+07 | -2.32E+07 | -3.43E+05 | -6.01E+07 | -1.92E+02 | 80 | 1.18E-06 |
| RelaxLn | 5e-7 log | 1.77E+09 | -1.77E+09 | -8.35E+07 | -8.32E+09 | -5.39E+03 | 86 | 3.55E-08 |
| RelaxLn | 5e-7 nor | 1.40E+09 | -1.40E+09 | -3.58E+07 | -1.45E+09 | -1.90E+04 | 275 | 7.00E-08 |
| RelaxLn | 5e-8 log | 3.86E+11 | -3.86E+11 | -1.26E+10 | -1.71E+12 | -1.50E+06 | 77 | 2.37E-10 |
| RelaxLn | 5e-8 nor | 3.65E+09 | -3.65E+09 | -6.80E+08 | -1.52E+10 | -1.11E+06 | 207 | 7.75E-10 |

Table A5: **Estimated root height and overall (averaged over all branches) mean clock rate for relaxed clocks with normal or log-normal priors on the rate of the relaxed clock (most variation)**

### **Estimation using tick samples only**

Tables A3-A5 and Figure A3 shows that considerable variation in the time-scale estimates is possible, depending on the model settings, especially the choice of clock rate priors. This is partly due to attempting to infer time-scales between integrated and non-integrated ASFV. Furthermore, the effective sample size (ESS) of the MCMC traces from these models were poor (despite running the chain for at least 10,000 steps which would normally be more than sufficient for six taxa), indicating poor model fit.

Therefore, we also inferred time-scaled trees using the five tick samples only. Here we expected that using a strict clock (no variation between branches) and a clock rate prior commensurate with the expected substitution rate for the host DNA would be most appropriate. The aligned nucleotide sequences were used to infer time-scaled trees (excluding the Ken06/Bus reference sequence), using a TN93 model with invariant sites and gamma-distributed rates for the variable sites (four categories), strict clock, constant population size, together with the 15 indel sites which differed between sequences 1-5 (final column of Table A2).

|  |  | **Root Height** | **Root Age statistics** | | | | | **Mean Rate** |
| --- | --- | --- | --- | --- | --- | --- | --- | --- |
| **Clock** | **Prior** | **mean** | **mean** | **median** | **95% Lower** | **95% Upper** | **ESS** | **mean** |
| Strict | 5e-6 log | 1.11E+04 | -9.13E+03 | -6.04E+03 | -2.73E+04 | 7.49E+02 | 298 | 2.46E-06 |
| Strict | 1e-6 log | 4.50E+04 | -4.30E+04 | -3.01E+04 | -1.25E+05 | -9.44E+02 | 378 | 6.85E-07 |
| Strict | 5e-7 log | 8.08E+04 | -7.88E+04 | -5.77E+04 | -2.18E+05 | -4.10E+03 | 377 | 3.55E-07 |
| Strict | 1e-7 log | 4.50E+05 | -4.48E+05 | -3.20E+05 | -1.25E+06 | -1.68E+04 | 364 | 7.02E-08 |
| Strict | 5e-8 log | 8.91E+05 | -8.89E+05 | -6.24E+05 | -2.48E+06 | -6.66E+04 | 377 | 3.36E-08 |
| Strict | 1e-8 log | 4.70E+06 | -4.70E+06 | -3.22E+06 | -1.31E+07 | -1.42E+05 | 391 | 7.00E-09 |

Table A6: Estimated root height and overall (averaged over all branches) mean clock rate for strict clocks with log-normal priors on the rate of the strict clock for the five ASFV-like sequences from tick samples only.

The difference in fit between these models and priors was assessed by estimating the marginal likelihood using path sampling and stepping stone sampling in BEAST. The results indicate that the log-normal clock rate prior with mean 5e-7 is best, however, the difference in log likelihoods is not very significant between the different prior settings, especially settings 1e-7, 5e-8 and 1e-8 (Table A7).

| **Clock** | **Prior** | **Path** | **Stepping** | **Diff Max-Path** | **Diff Max-Stepping** |
| --- | --- | --- | --- | --- | --- |
| Strict | 5e-6 log | -2797.86 | -2797.90 | 0.93 | 0.95 |
| Strict | 1e-6 log | -2798.40 | -2798.53 | 1.47 | 1.58 |
| Strict | 5e-7 log | -2796.93 | -2796.95 | 0.00 | 0.00 |
| Strict | 1e-7 log | -2797.23 | -2797.31 | 0.30 | 0.36 |
| Strict | 5e-8 log | -2797.44 | -2797.51 | 0.51 | 0.56 |
| Strict | 1e-8 log | -2797.81 | -2797.91 | 0.88 | 0.97 |

Table A7: Marginal likelihood estimation using Path Sampling and Stepping Stone Sampling, showing that the log-normal clock rate prior with mean = 5e-7 is best, but not significantly better than the other clock rates.


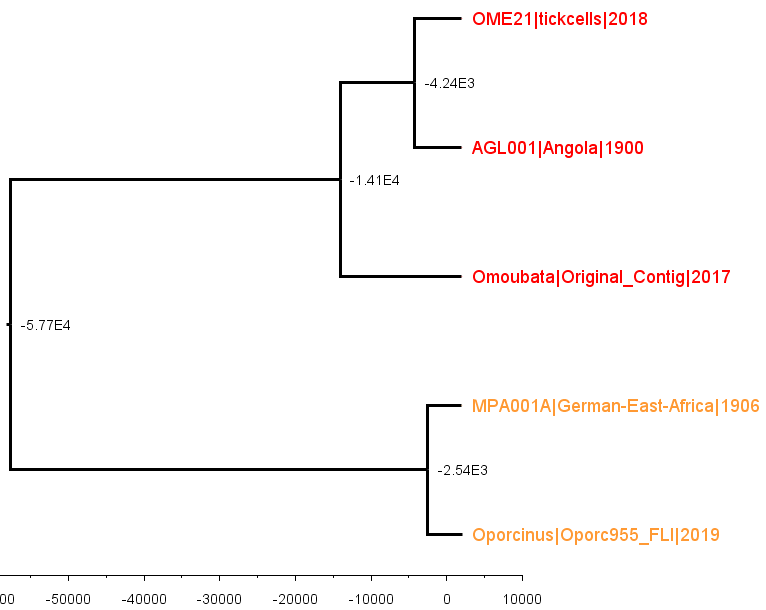


Figure A4: **Time-scaled tree for partial EP1242L sequences from tick samples only (5e-7 substitutions per site per year).** The tree was inferred using a strict molecular clock and log-normal clock rate prior with mean and standard deviation of 5e-7 substitutions per site per year. Node ages are indicated in years BCE.


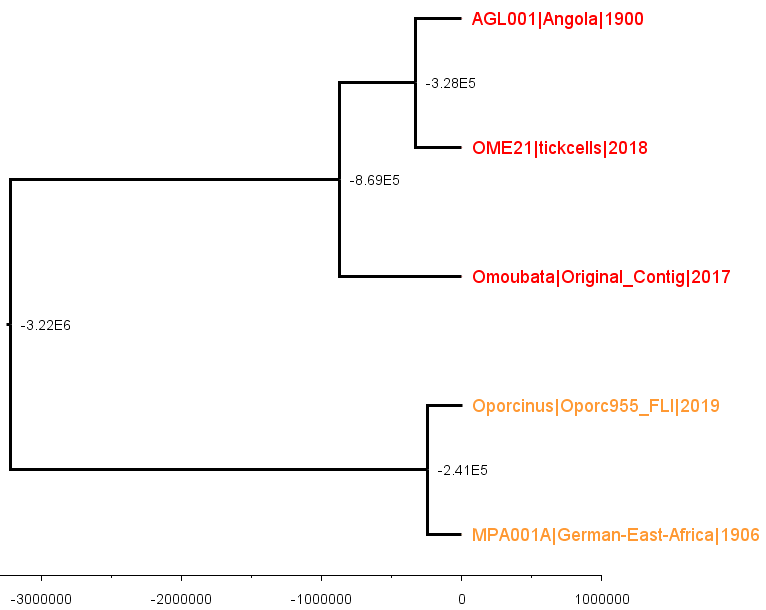


Figure A5: **Time-scaled tree for partial EP1242L sequences from tick samples (1e-8 substitutions per site and year).** The tree was inferred using a strict molecular clock and log-normal clock rate prior with mean and standard deviation of 1e-8 substitutions per site and year. Node ages are indicated in years BCE.
