# Supplementary figures and images for: Identification of African swine fever virus-like elements in the soft tick genome provides insights into the virus’ evolution
Source: BMC Biol. 2020 Oct 8;18:136. doi: 10.1186/s12915-020-00865-6 (PMC7542975; doi:10.1186/s12915-020-00865-6)

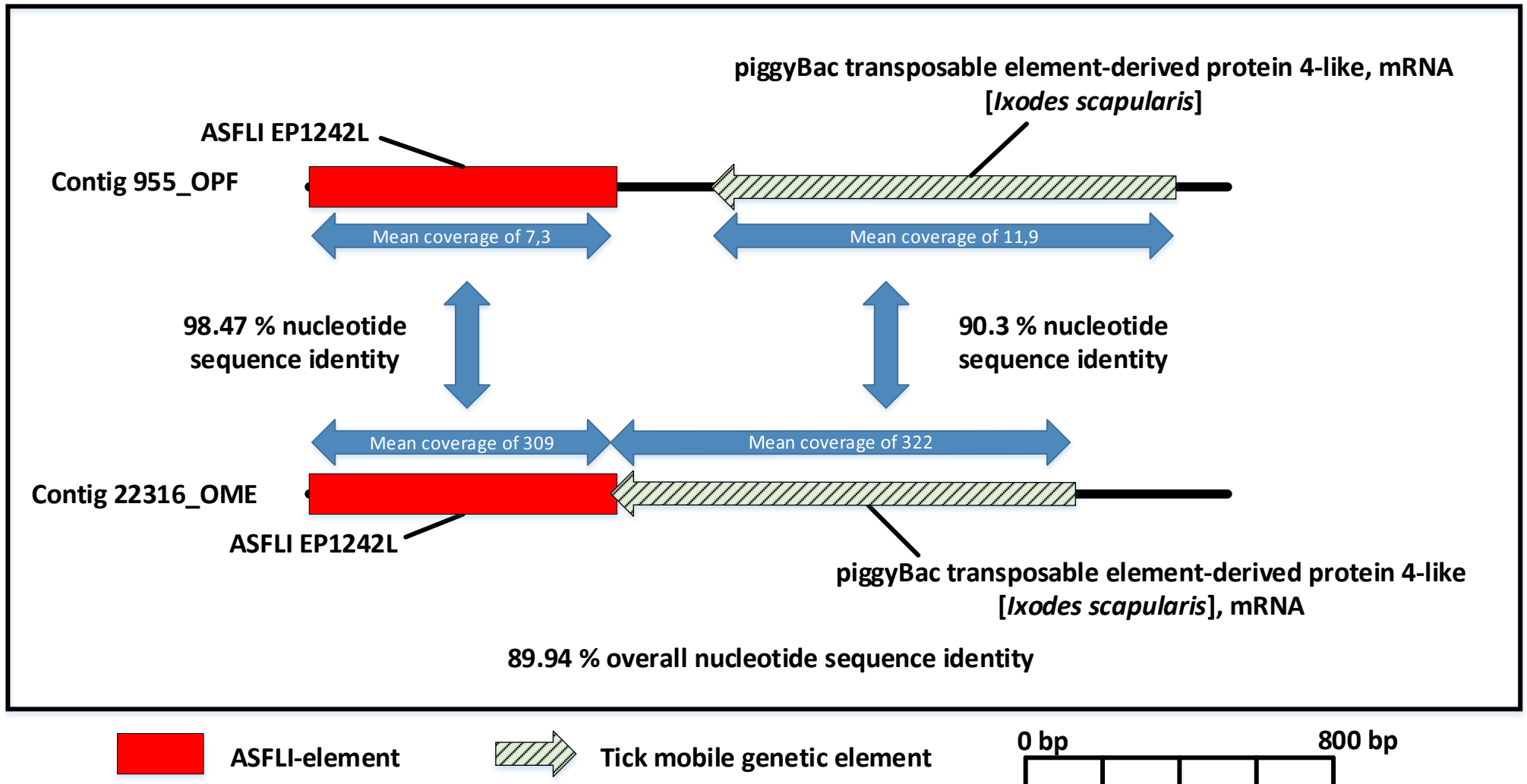

Supplement: Supplementary file 8 — Additional file 8: Figure S1.Similarity between ASFLI-element contigs identified in Ornithodoros moubata cells and Ornithodoros porcinus ticks. Contigs were assembled using SPAdes 3.13. on sequencing data obtained from O. porcinus ticks (OPF) and and OME/CTVM21 cells (OME) using default parameters. After contig identification by BLASTn and BLASTp search against the entire NCBI database and annotation, contigs were aligned using MAFFT v7.388 in Geneious. To analyse the possible existence of multiple copies of the piggyback transposable element, coverage was calculated in Geneious from mapping of libs 02151-2 and lib02339 against Contig 22316_OME and lib03101-2 against Contig 955_OPF using Bowtie2 with default paramteres in the “very sensitive” mode. [file 12915_2020_865_MOESM8_ESM.pdf]

A

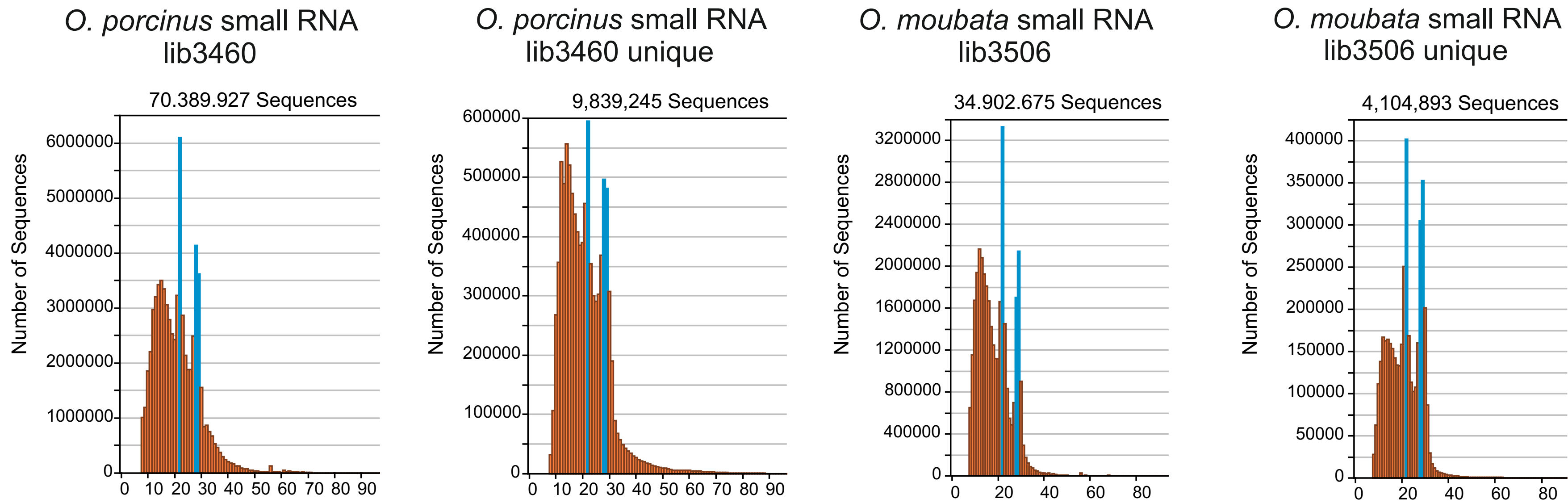

B

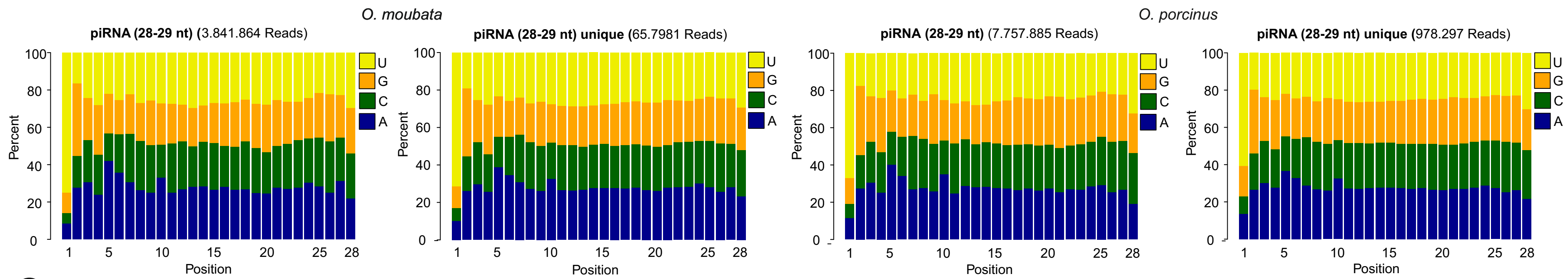

C

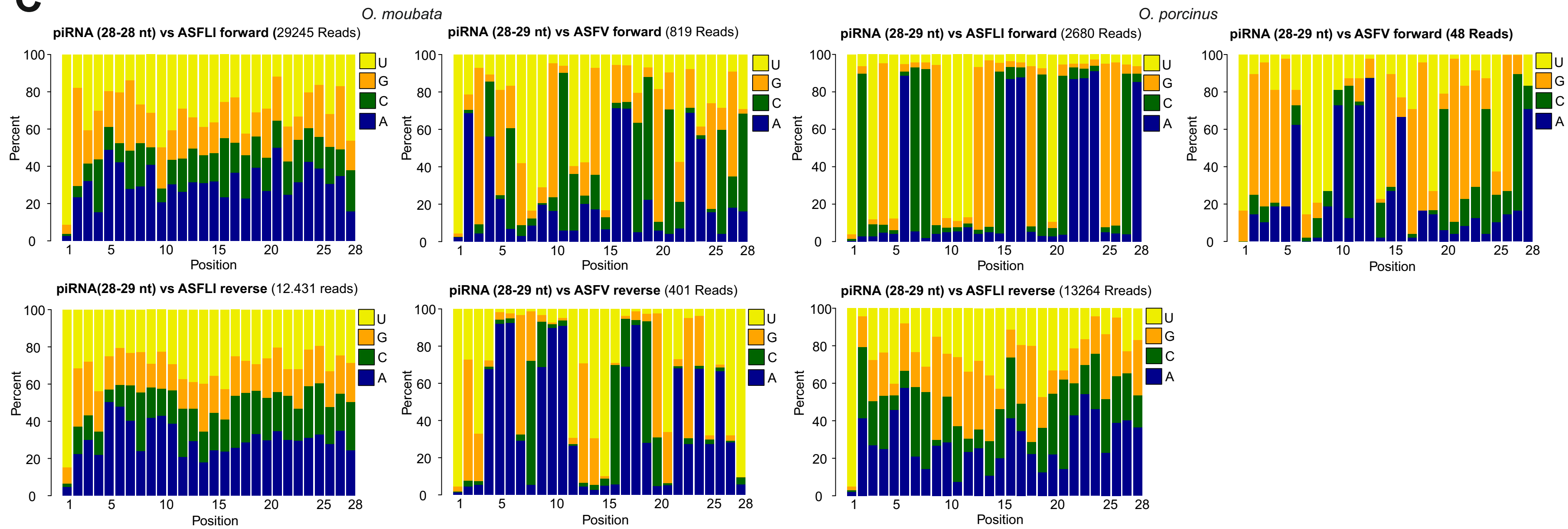

Supplement: Supplementary file 11 — Additional file 11: Figure S2. Read length distribution and piRNA nucleotide bias of small RNA libraries. The length distribution is shown from small RNA libraries before and after removal of duplicate reads. Blue colored bars indicate siRNA (22nt) and piRNA (28-29nt) fractions (A). Reads of the piRNA fraction (28-29nt) were extracted from raw and deduplicated libraries, trimmed at the 3’ end to 28 nt for analysis and nucleotide frequencies for every position were calculated using R-studio (https://www.rstudio.com) for the entire dataset (B) and for non-deduplicated reads mapped to all ASFLI-elements and all available ASFV whole-genome sequences using Bowtie in Geneious with 28 nt seed length and one allowed missmatch (C). piRNA from O. porcinus did only map to the ASFV forward strand. [file 12915_2020_865_MOESM11_ESM.pdf]

*O. moubata*

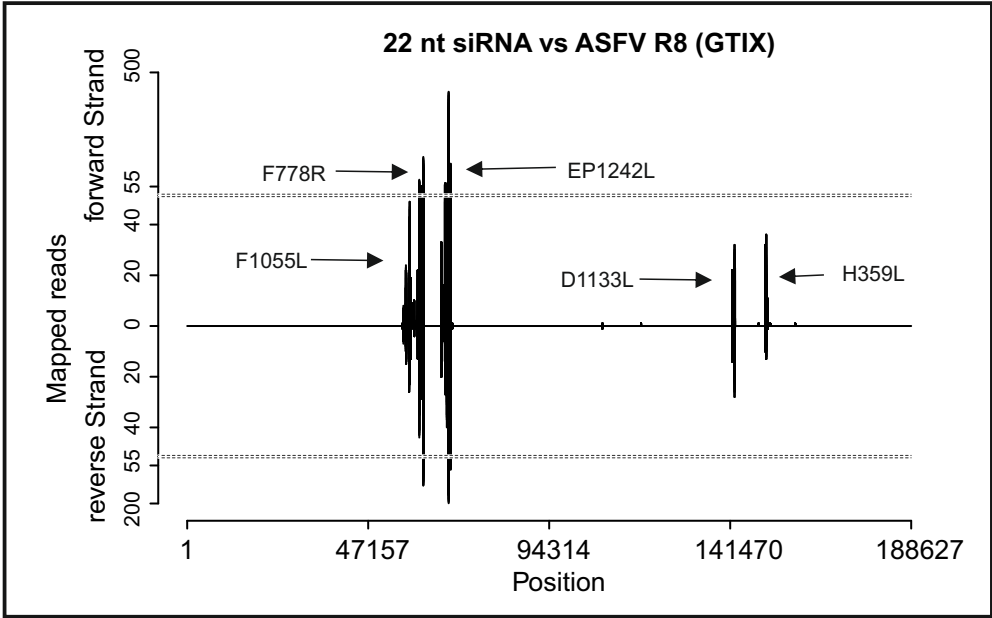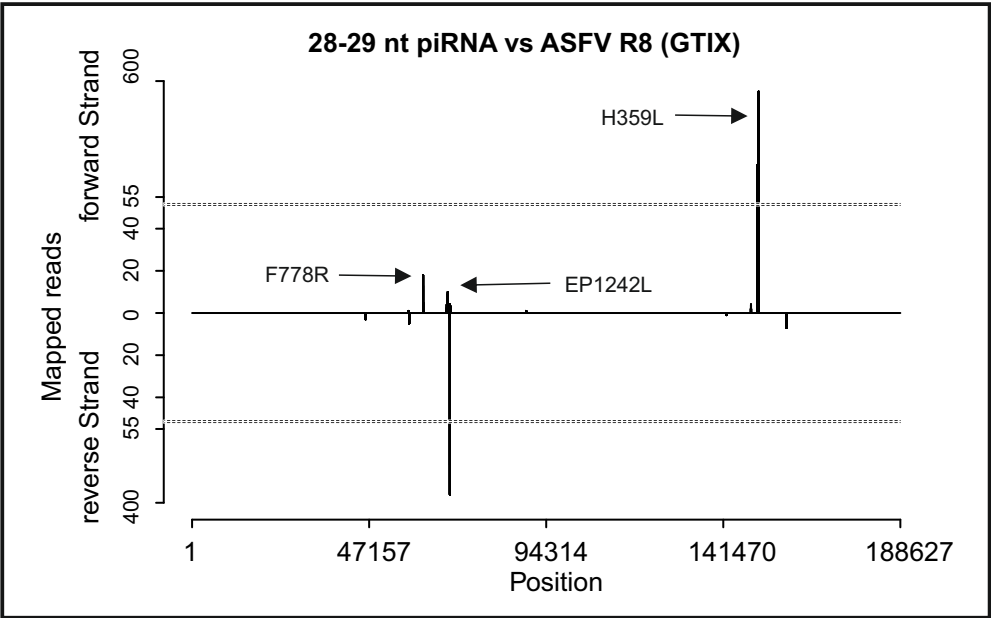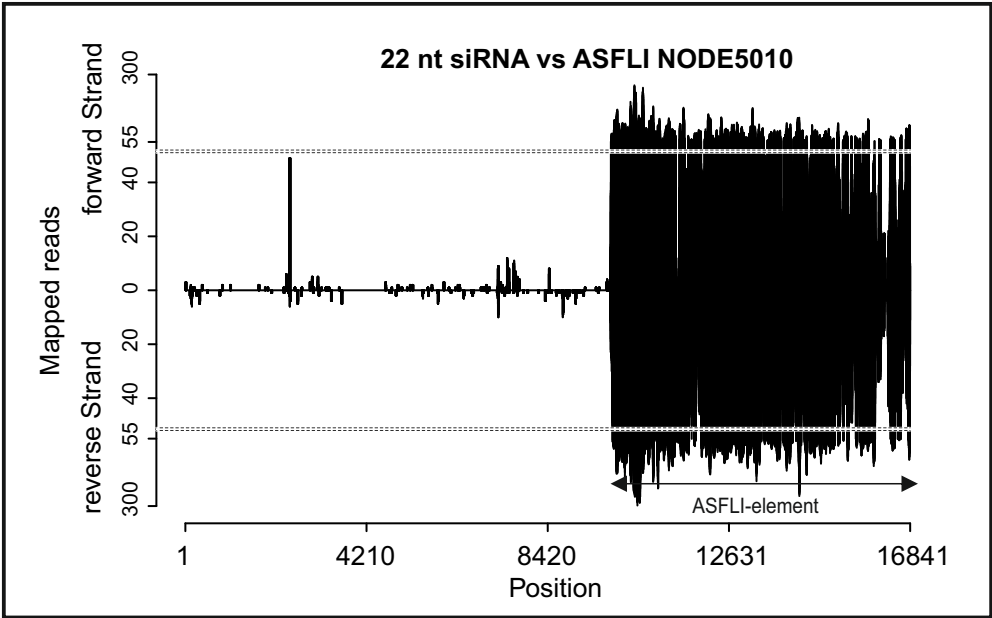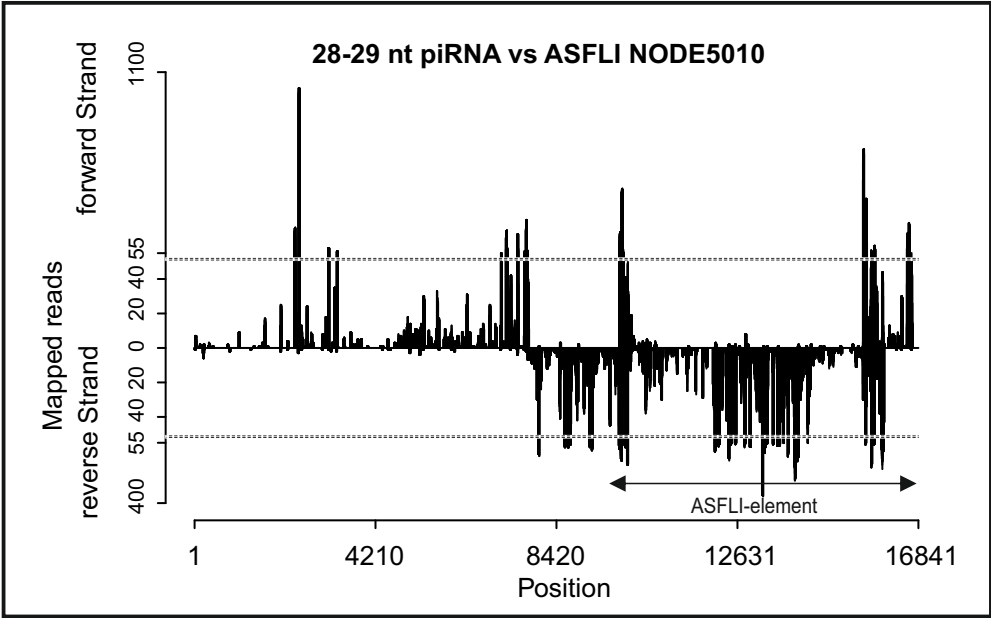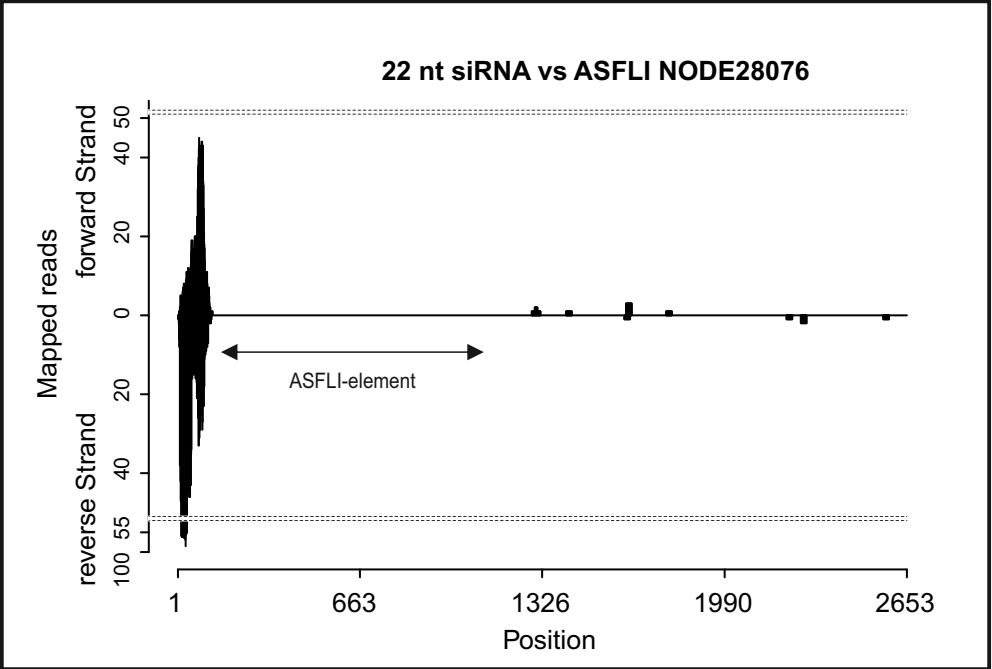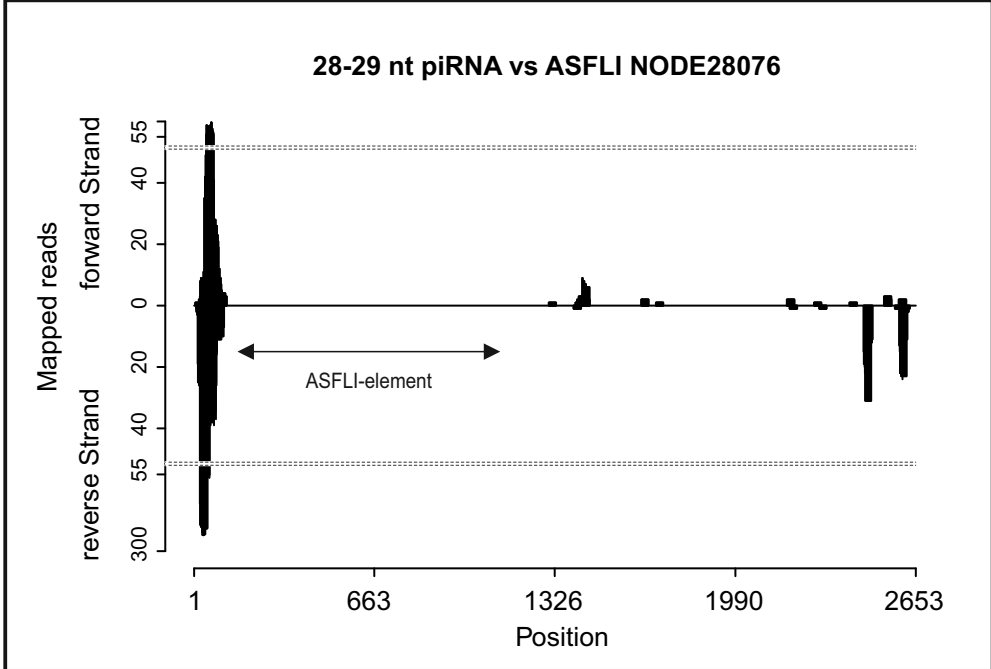

Supplement: Supplementary file 12 — Additional file 12: Figure S3. siRNA/piRNA mapping against ASFV-R8 (G IX) and two ASFLI-elements. siRNA (22 nt) and piRNA (29 nt) fractions from Ornithodoros moubata were individually mapped against available ASFV whole-genome sequences (the one showing the most mapped reads, ASFV-R8 (GT IX) is shown) and two exemplary O. moubata ASFLI-element-containing contigs (NODE5010 and NODE28076) using Bowtie (1.1.2) in Geneious. Marked (arrows) are the most abundant small RNA molecules related to the ASFV genes and ASFLI-elements. [file 12915_2020_865_MOESM12_ESM.pdf]

*O. porcinus*

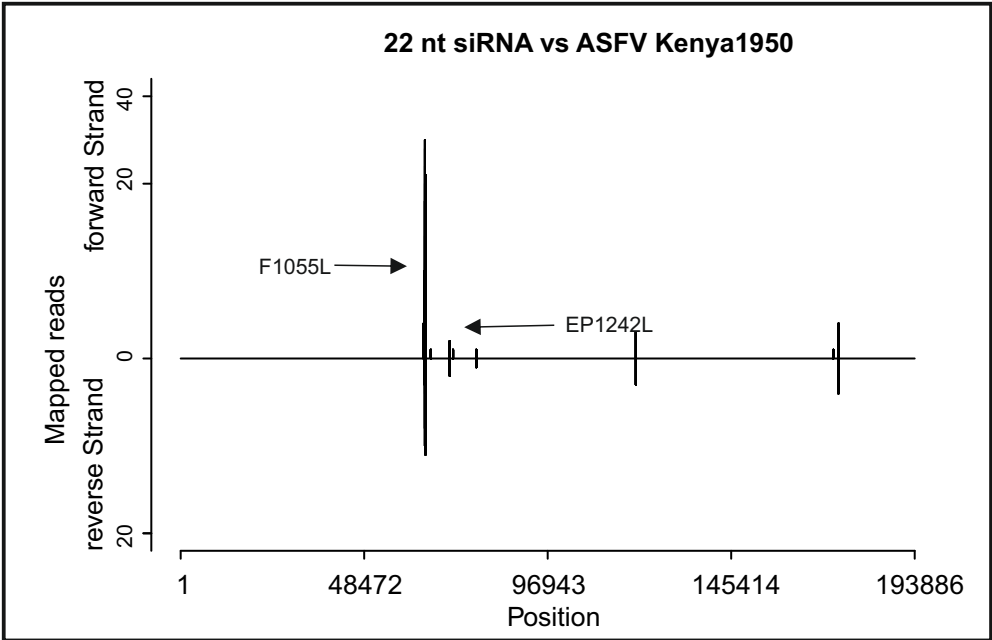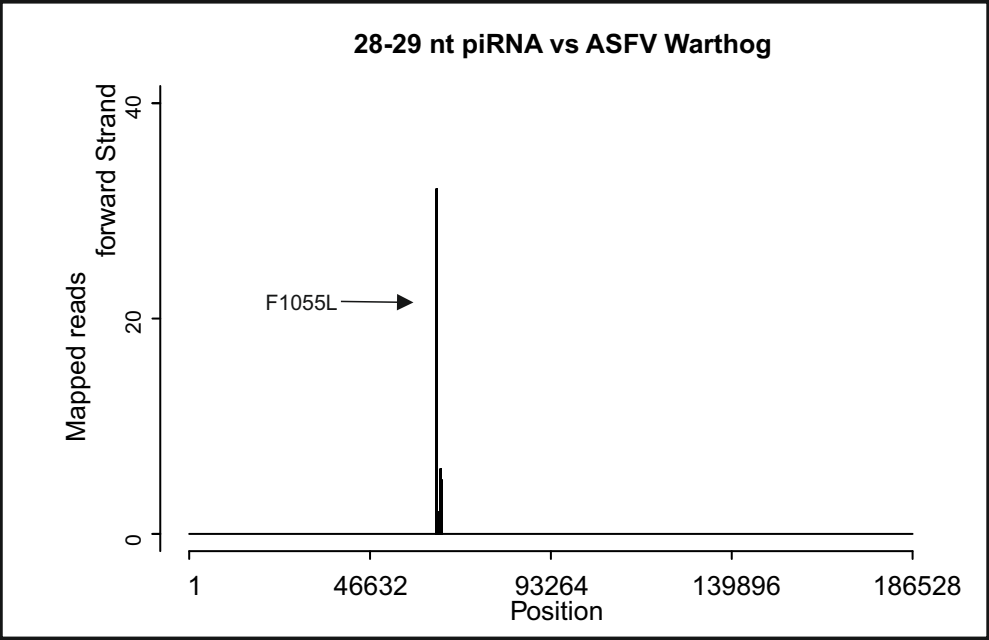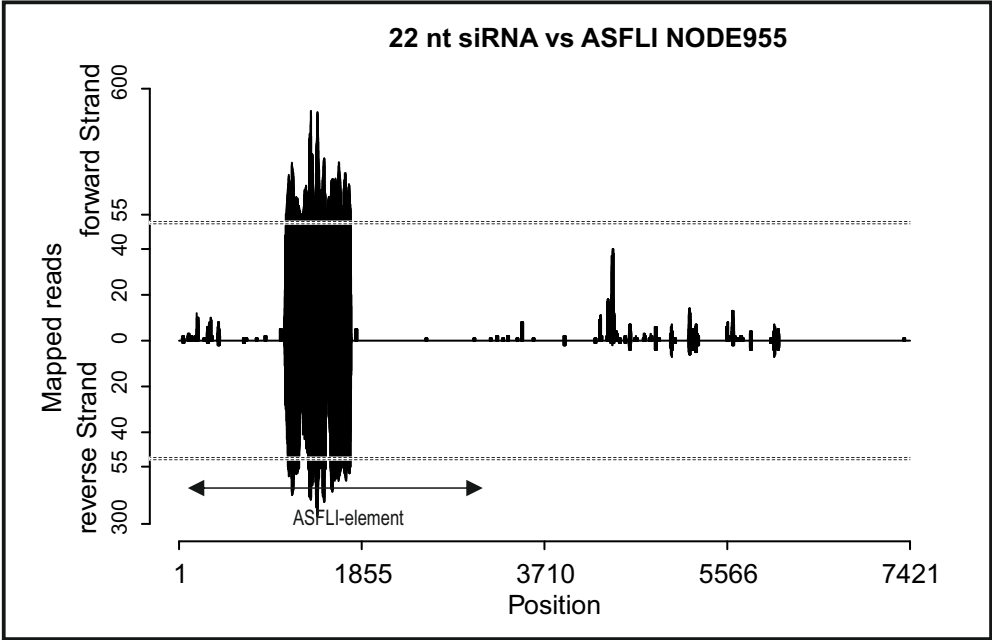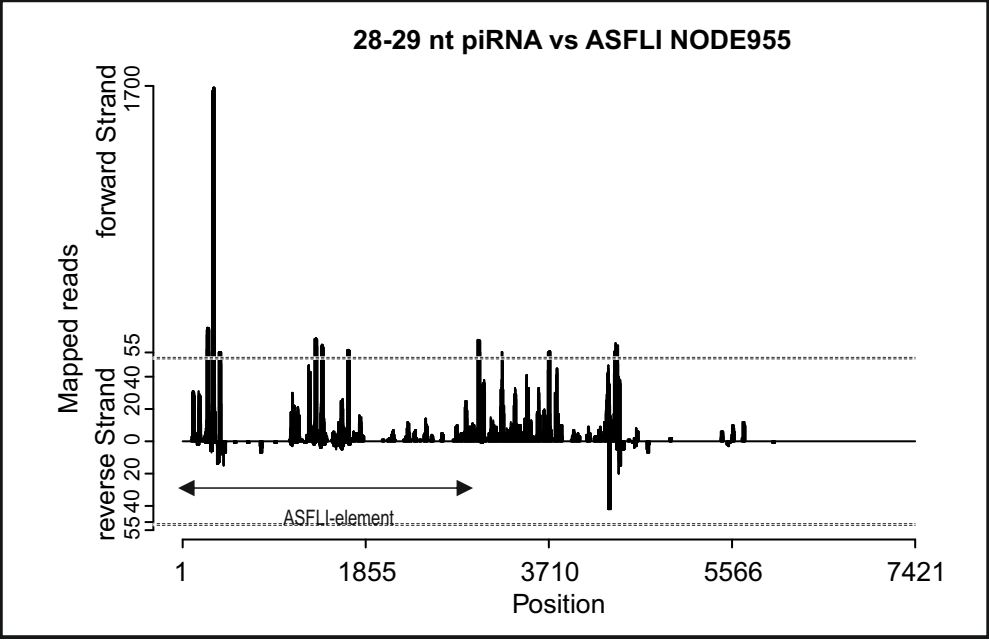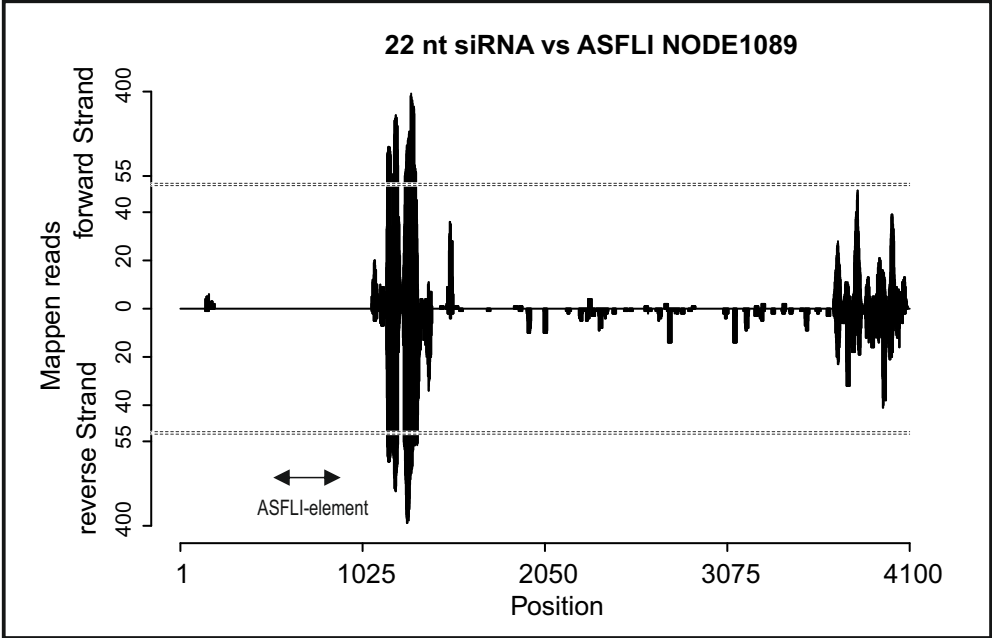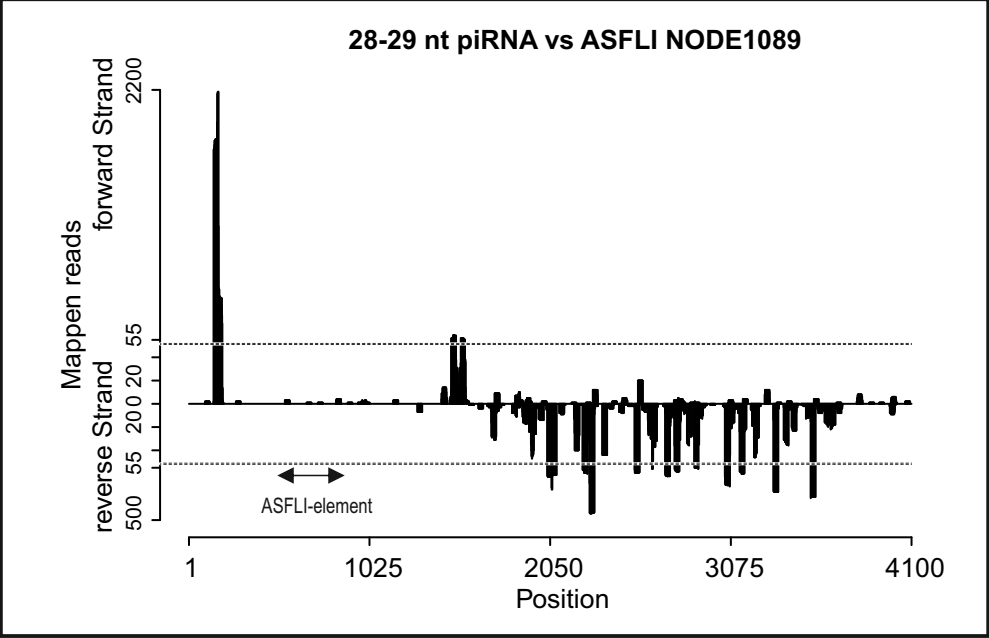

Supplement: Supplementary file 13 — Additional file 13: Figure S4. siRNA/piRNA mapping against ASFV-Kenya1950 (GT X), Warthog (G IV) and two ASFLI-elements. siRNA (22 nt) and piRNA (28-29 nt) fractions from Ornithodoros porcinus were individually mapped against available ASFV whole-genome sequences (the ones showing the most mapped reads, ASFV-Kenya1950 (GT X), ASFV Wathog (GT IV) are shown) and two exemplary O. porcinus ASFLI-element containing contigs (NODE955 and NODE1089) using Bowtie 1 (1.1.2) in Geneious. Marked (arrows) are the most abundant small RNA molecules relating to the ASFV genes and ASFLI-elements. [file 12915_2020_865_MOESM13_ESM.pdf]

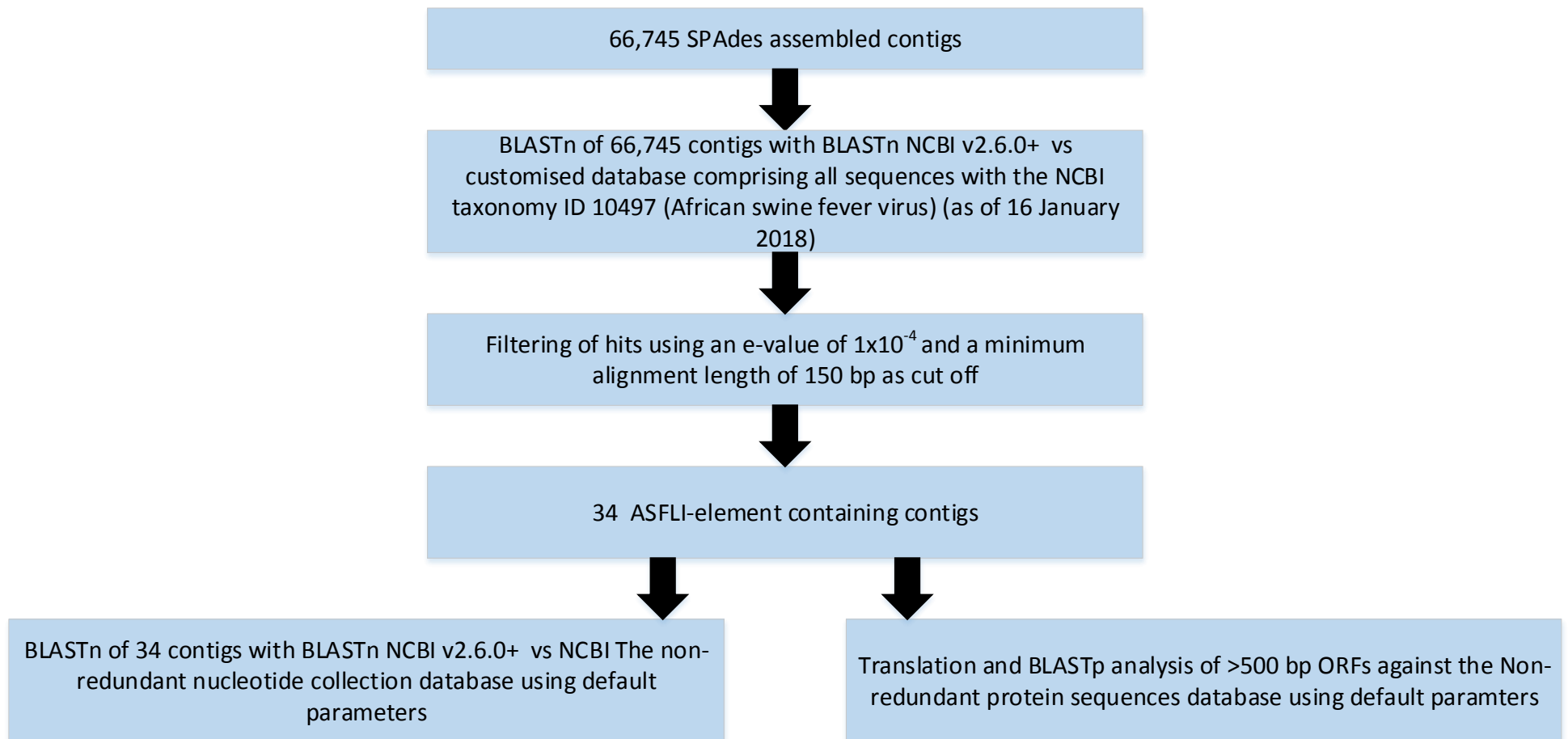

Supplement: Supplementary file 20 — Additional file 20: Figure S7. BLAST-analysis for identification of ASFLI-element containing contigs and annotation. 66,745 SPAdes assembled contigs were blasted (BLASTn, NCBI, v2.6.0+) against a customised database comprising all sequences with the NCBI taxonomy ID 10497 (African swine fever virus) (as of 16 January 2018). Hits were filtered using a cut off e-value of 1x10^-4^ and a minimum alignment length of 150 bp, resulting in 34 contigs. These were then blasted against the complete NCBI database (The non-redundant nucleotide collection) to reliably identify and annotate ASFV-like sequences and areas of the host genome using default parameters. BLASTp search of >500 bp ORFs was performed against the “Non-redundant protein sequences” database using default parameters. [file 12915_2020_865_MOESM20_ESM.pdf]
